# Structural details of amyloid β oligomers in complex with human prion protein as revealed by solid-state MAS NMR spectroscopy

**DOI:** 10.1016/j.jbc.2021.100499

**Published:** 2021-03-03

**Authors:** Anna S. König, Nadine S. Rösener, Lothar Gremer, Markus Tusche, Daniel Flender, Elke Reinartz, Wolfgang Hoyer, Philipp Neudecker, Dieter Willbold, Henrike Heise

**Affiliations:** 1 Institute of Biological Information Processing (IBI-7: Structural Biochemistry) and JuStruct: Jülich Center for Structural Biology, Forschungszentrum Jülich, Jülich, Germany; 2 Institut für Physikalische Biologie, Heinrich-Heine-Universität Düsseldorf, Düsseldorf, Germany; 3 Research Center for Molecular Mechanisms of Aging and Age-Related Diseases, Moscow Institute of Physics and Technology (State University), Dolgoprudny, Russia

**Keywords:** Alzheimer disease, amyloid beta (Aβ), prion protein, oligomer, solid-state NMR, solution NMR, nuclear magnetic resonance (NMR), structural biology, Aβ, amyloid β, AD, Alzheimer’s disease, CP, cross polarization, DE, direct excitation, INEPT, insensitive nuclei enhancement by polarizaiton transfer, PDSD, proton-driven spin-diffusion, PrP^C^, cellular prion protein, RCI, Random Coil Index

## Abstract

Human PrP (huPrP) is a high-affinity receptor for oligomeric amyloid β (Aβ) protein aggregates. Binding of Aβ oligomers to membrane-anchored huPrP has been suggested to trigger neurotoxic cell signaling in Alzheimer’s disease, while an N-terminal soluble fragment of huPrP can sequester Aβ oligomers and reduce their toxicity. Synthetic oligomeric Aβ species are known to be heterogeneous, dynamic, and transient, rendering their structural investigation particularly challenging. Here, using huPrP to preserve Aβ oligomers by coprecipitating them into large heteroassemblies, we investigated the conformations of Aβ(1–42) oligomers and huPrP in the complex by solid-state MAS NMR spectroscopy. The disordered N-terminal region of huPrP becomes immobilized in the complex and therefore visible in dipolar spectra without adopting chemical shifts characteristic of a regular secondary structure. Most of the well-defined C-terminal part of huPrP is part of the rigid complex, and solid-state NMR spectra suggest a loss in regular secondary structure in the two C-terminal α-helices. For Aβ(1–42) oligomers in complex with huPrP, secondary chemical shifts reveal substantial β-strand content. Importantly, not all Aβ(1–42) molecules within the complex have identical conformations. Comparison with the chemical shifts of synthetic Aβ fibrils suggests that the Aβ oligomer preparation represents a heterogeneous mixture of β-strand-rich assemblies, of which some have the potential to evolve and elongate into different fibril polymorphs, reflecting a general propensity of Aβ to adopt variable β-strand-rich conformers. Taken together, our results reveal structural changes in huPrP upon binding to Aβ oligomers that suggest a role of the C terminus of huPrP in cell signaling. Trapping Aβ(1–42) oligomers by binding to huPrP has proved to be a useful tool for studying the structure of these highly heterogeneous β-strand-rich assemblies.

Alzheimer’s disease (AD) accounts for an estimated 60 to 80% of all types of dementia (1). One of the hallmarks of AD is the formation of amyloid plaques, which consist mainly of amyloid β (Aβ) peptides comprising 39 to 43 residues (2). Aβ is produced by cleavage of the amyloid precursor protein (APP) by β- and γ-secretases (3). Of the two most abundant species Aβ(1–40) and Aβ(1–42), the latter is more prone to aggregation and its aggregates are more toxic (3). Small to moderately sized Aβ oligomers (Aβ_oligos_) have been identified as the most neurotoxic factor in the pathogenesis of AD, whereas large fibrils are known to be the main component of insoluble plaques (4). Detailed structural information on Aβ(1–42)_oligo_ is thus of paramount interest, and in recent years, structural studies on different oligomer preparations of Aβ(1–42)_oligo_, Aβ(1–40)_oligo_ (5, 6) (or pyro-Glu-Aβ(3/11–40) oligomers (7)) by solid-state NMR-spectroscopy have been conducted (8, 9, 10, 11, 12, 13, 14, 15). Shape, morphology, and structural details of those oligomers were strongly dependent on preparation conditions, and while all of these oligomers had a high prevalence of β-strand secondary structure, tertiary fold and supramolecular arrangement of β-strands were found to differ strongly between different preparations. While in most mature fibrils β-strands are arranged in parallel in-register β-sheets (16, 17), quaternary structures in oligomers are much more variable, and, depending on the fibrillation pathway, parallel (12), antiparallel (18) β-sheets or even a mixture of both (11) have been found. A major challenge to structural studies of oligomers is their transient nature, and thus, most oligomer preparations exhibit substantial structural heterogeneity. Stabilization of oligomers is essential for long-term structural investigations. In most cases, further aggregation of oligomers was prevented by freeze-trapping with subsequent lyophilization (7, 8, 9, 10, 11, 12, 14). In this study, we used the recombinant human prion protein in its native cellular prion protein (PrP^C^) conformation to trap Aβ oligomers by coprecipitating them into large heteroassemblies, in which the growth of Aβ_oligo_ is prevented, as demonstrated by long-term solid-state NMR measurements over 11 months.

The PrP^C^ is a high-affinity cell-surface receptor for Aβ_oligo_ (19, 20), and PrP^C^ is also able to bind to fibrillar Aβ (21, 22, 23). It has been suggested that binding of Aβ_oligo_ to membrane-anchored PrP^C^ mediates Aβ toxicity during AD by mediating synapse damage (24) and the blockade of long-term potentiation by Aβ_oligo_ (19, 25) *via* activation of Fyn-kinase pathways (26, 27) (Fig. S1), but this has also been questioned (28, 29, 30, 31). It has also been described that soluble PrP (32) and its N-terminal fragment PrP(23–111) (33, 34) have a protective role by inhibiting Aβ fibrillation and sequestration of Aβ_oligo_.

Several *in vitro* studies on the Aβ-PrP interaction suggest that Aβ_oligos_ bind at two Lys-rich parts (residues 23–27 and ≈95–110) on PrP (35, 36, 37, 38, 39, 40), but an additional involvement of the C terminus of PrP has also been suggested (21). Interestingly, the N terminus of human PrP is also able to bind oligomeric α-synuclein with high affinity (41, 42, 43). A structural study of insoluble PrP^C^-Aβ_oligo_ complexes described them as a “hydrogel,” in which the Aβ(1−42)_oligos_ were rigid, while PrP still has high molecular mobility (44). Additionally, this study reported a conformational change in the N terminus of PrP^C^ upon complexation with Aβ_oligo_. We recently demonstrated that Aβ_oligo_ forms large heteroassemblies with either full-length (huPrP(23–230)) or C-terminally truncated (huPrP(23–144)) membrane-anchorless monomeric PrP (40). These assemblies have a size of a few micrometers as determined by dynamic light scattering and show cloud-like morphologies as seen by atomic force microscopy (40). The Aβ:huPrP stoichiometry of the heteroassemblies depends on the amount of huPrP added to Aβ_oligo_ and reaches a value of 4:1 (monomer ratio Aβ:huPrP) if either huPrP(23–144) or huPrP(23–230) is added to the oligomer solution in excess (40). In all these *in vitro* preparations, Aβ oligomers and early-stage protofibrils are stabilized and prevented from elongation by PrP, which has been shown to preferentially bind to fast-growing fibril and oligomer ends (22).

Here we exploit this stabilizing effect in an NMR study on different samples of Aβ_oligo_ complexed by huPrP. Isotope labeling of either huPrP or Aβ allowed us to characterize both components of the complex separately. While the N-terminal region of huPrP in the complex remains largely devoid of secondary structure and still undergoes fast backbone conformational averaging on the microsecond to millisecond timescale, Aβ_oligos_ exhibit a high degree of β-strand conformation. While these Aβ_oligos_ are highly heterogeneous, solid-state NMR spectra reveal similarities with the corresponding spectra of all fibril polymorphs published so far (45, 46, 47).

## Results

### The N-terminal construct huPrP(23–144) is disordered in solution at mildly acidic and neutral pH

The solution structure of huPrP(23–230) had originally been determined in acetate buffer at an acidic pH of 4.5 and a temperature of 20 °C (48), whereas the huPrP-Aβ(1–42)_oligo_ complex samples for solid-state NMR were prepared at a pH value close to neutral. As a basis for studying the interaction between huPrP and Aβ_oligo_, we therefore first investigated free huPrP(23–144) by NMR spectroscopy in solution at different pH values ranging from 4.5 to 7.0 and at a temperature of 5.0 °C, which is closer to the temperature used for the solid-state NMR experiments. As reported previously, the chemical shifts of the N-terminal amino acid residues 23 to 124 in truncated huPrP(23–144) are almost identical to those of huPrP(23–230), whereas residues 125 to 144, which are part of the well-ordered globular domain of huPrP(23–230), are strongly affected by the truncation at position 144 (40).

We obtained almost complete sequence-specific ^1^H, ^13^C, and ^15^N backbone resonance assignments for huPrP(23–144) at pH values of 4.5 and 7.0 and a temperature of 5.0 °C using a combination of HNCO, HNCACB, and BEST-TROSY-(H)N(COCA)NH triple-resonance experiments (Fig. S2). The assigned chemical shifts at pH 4.5 and pH 7.0 have been deposited with the Biological Magnetic Resonance Data Bank (BMRB) under accession codes 28115 and 28116, respectively.

As expected, side-chain titration in this pH range causes significant chemical shift changes for all seven histidine residues and for residues next to histidine. Other than that, the chemical shifts at pH 4.5 and pH 7.0 are very similar to each other and very close to random coil shifts (49). Quantitative analysis reveals that the Random Coil Index (RCI) order parameters (50) S_RCI_^2^, which are a measure of how different the backbone chemical shifts are from those of a disordered random coil on a scale of 0 (typical for a random coil) to 1 (typical for a well-ordered backbone conformation), are consistently below ≈0.6 (Fig. S3). This demonstrates conclusively that free huPrP(23–144) in solution at neutral and mildly acidic pH is highly disordered and devoid of any stable secondary structure.

### The flexible N terminus of huPrP becomes immobilized but remains almost devoid of regular secondary structure upon binding to Aβ_oligo_

High-molecular-weight heteroassemblies of oligomeric Aβ(1–42) and huPrP(23–144) or of oligomeric Aβ(1–42) and huPrP(23–230) were prepared by adding the respective huPrP construct to a preincubated solution of Aβ(1–42), as described previously (40). Immediately after addition of huPrP to the solution, precipitation of a solid fine white powder was observed.

**Figure 1:**
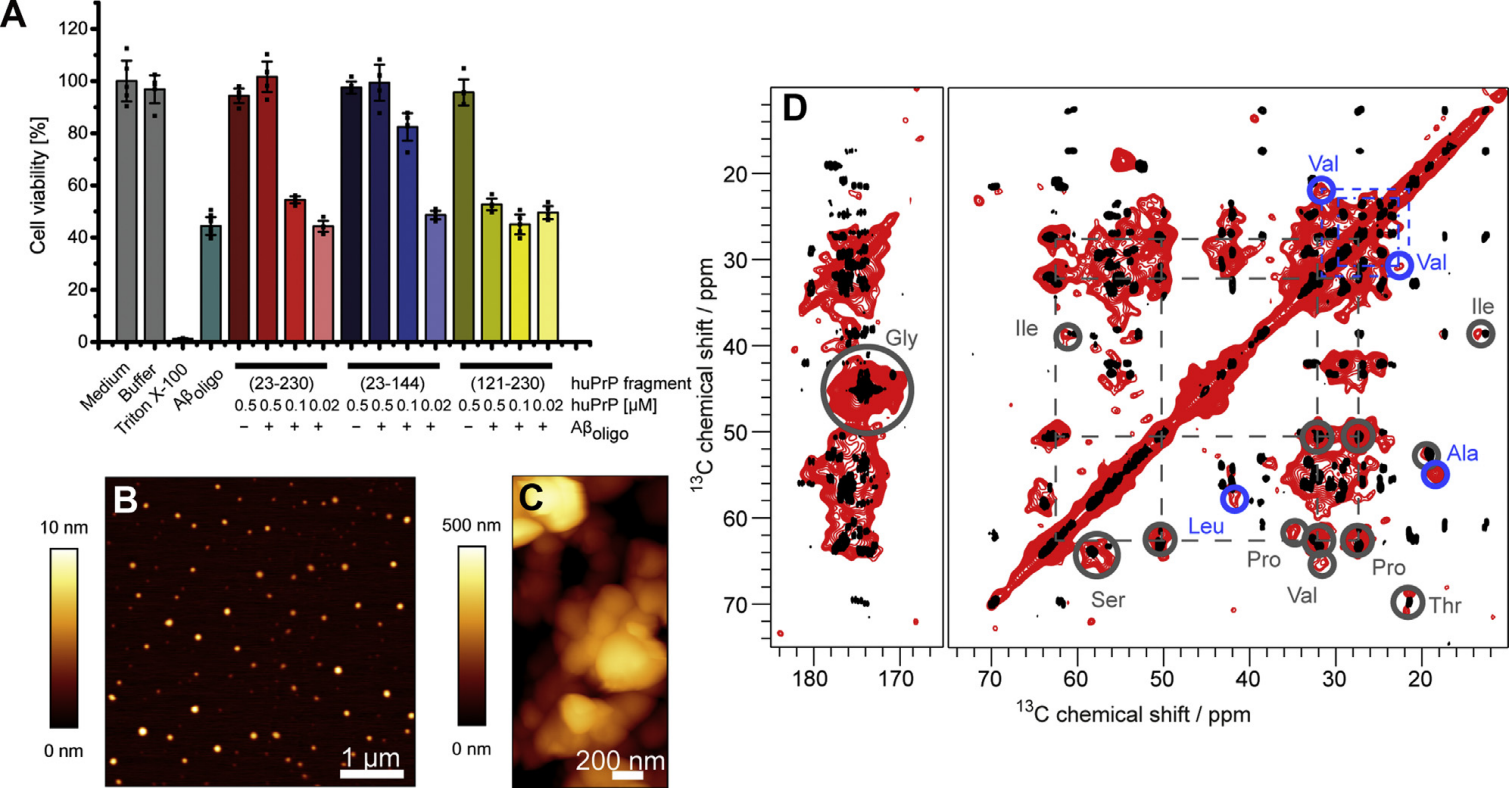
***A*, MTT assay of 1 μM Aβ**_**oligo**_**and of 1 μM Aβ**_**oligo**_**in complex with either 0.5 μM, 0.1 μM, or 0.02 μM of either huPrP(23–230), huPrP(23–144) or huPrP(121–230).** Both huPrP(23–230) and huPrP(23–144) reduce Aβ_oligo_ toxicity in a concentration-dependent manner. In contrast, the C-terminal fragment huPrP(121–230) does not. None of the huPrP fragments alone reduces cell viability. This reduction of toxicity has been seen for non-membrane-bound huPrP fragments before (32, 36) and is in contrast to toxic effects of membrane-anchored huPrP (19, 24, 25). As our complexes do not exhibit a GPI-anchor, the reduction of toxicity reflects these observations. *B*, 5 μm × 5 μm AFM image of 440 nM Aβ_oligo_ and *C*, 2 μm × 1 μm AFM image of Aβ_oligo_-huPrP(23–144) coprecipitates generated with 80 μM preincubated Aβ(1–42) and 40 μM huPrP(23–144). The aggregates have sizes up to 1 μm spanning clusters with a smooth surface appearance, whereas Aβ_oligo_ are small nm spheres. *D*, comparison of a PDSD spectrum of huPrP(23–144)∗-Aβ (∗ species is ^13^C, ^15^N uniformly labeled) in *red* with a ^13^C-^13^C TOCSY spectrum of monomeric huPrP(23–144) in *black*. The PDSD spectrum was recorded at a temperature of ≈−6 °C, a spinning frequency of 11 kHz and a mixing time of 30 ms and the TOCSY spectrum at a temperature of 5.0 °C, at pH 6.7. *Gray circles* indicate some identified amino acid types, *dashed lines* Pro and Val connections in the PDSD spectrum. Due to broad line widths and a low signal dispersion in the PDSD spectrum several correlations overlap, especially for the residues in the octarepeat region. Nevertheless, spin systems for most of the amino acid types present in the sequence could be identified and an amino-acid-type specific resonance assignment was possible. Differences between the PDSD and TOCSY spectrum are highlighted with *blue circles*. For an additional PDSD spectrum see Fig. S6, the corresponding double quantum-single quantum correlation spectrum (DQ-SPC5) is shown in Fig. S7.

These formed high-molecular-weight heteroassemblies were analyzed by an MTT cell viability test (Fig. 1*A*). Both huPrP(23–230) and huPrP(23–144) reduce Aβ(1–42)_oligo_ toxicity in a concentration-dependent manner, thus these complexes are not toxic, and huPrP has a protective effect. As our complexes do not exhibit a GPI anchor, this fits to the observation of a protective role for non-membrane-bound huPrP fragments (32, 36) in contrast to membrane-anchored huPrP, which mediates neurotoxicity (19, 24, 25). The fragment huPrP(121–230), which was shown to not form any heteroassemblies (40), however, does not rescue Aβ(1–42)_oligo_ toxicity and was used as a negative control. None of the huPrP fragments alone is toxic for the cells (Fig. 1*A*).

High-molecular-weight assemblies of Aβ(1–42)_oligo_ and huPrP(23–144) were further analyzed by sucrose density gradient ultracentrifugation (DGC) and subsequent SDS-PAGE and RP-HPLC (40) (Fig. S4). As previously described (40), a molar ratio of Aβ:PrP of 4:1 is obtained in the assemblies if huPrP is added in excess; for higher Aβ:PrP ratios, not all potential PrP-binding sites on Aβ_oligo_ are saturated with huPrP(23–144) (as in sample huPrP(23–144)-Aβ∗, ∗ indicates that the Aβ moiety of the complex is ^13^C, ^15^N labeled).

In Figure 1 typical AFM images of Aβ_oligo_ alone (Fig. 1*B*) or in complex with N-terminal huPrP(23–144) (Fig. 1*C*) are shown. Spherical Aβ oligomers can clearly be identified (Fig. 1*B*), and no fibrils are observed in the huPrP(23–144)-Aβ condensates (Fig. 1*C*). Next, we focused on investigating structural features of the complex by NMR spectroscopy.

To probe the flexibility of the N-terminal construct huPrP(23–144) in the complex, we recorded a ^1^H-^13^C insensitive nuclei enhanced by polarization transfer (INEPT)-NMR spectrum as well as dipolar-based ^1^H-^13^C and ^1^H-^15^N cross polarization (CP)-MAS spectra (51). The INEPT-NMR spectrum of this sample did not show any protein signals at a sample temperature of ≈27 °C (spectrum not shown), whereas in ^1^H-^13^C (recorded at a sample temperature of ≈0 °C) and ^1^H-^15^N CP spectra (recorded at a sample temperature of ≈−6 °C) strong signals typical for all amino acid types can be seen (Fig. S5). This indicates that huPrP(23–144) in complex with Aβ(1–42)_oligo_ is immobilized and does not undergo rapid isotropic reorientation as in solution.

In Figure 1*D* a typical 2D ^13^C-^13^C-correlation spectrum obtained with proton-driven spin-diffusion (PDSD) of huPrP(23–144)∗-Aβ (∗ indicates that the huPrP moiety of the complex is ^13^C, ^15^N labeled) is overlaid with a ^13^C-^13^C total correlation spectrum (TOCSY) of monomeric huPrP(23–144) in solution at pH 6.7. Except for some Val and Ala resonances, most of the peaks align well. This indicates that the natively unfolded N terminus of huPrP does not undergo a major conformational rearrangement upon complex formation with Aβ_oligo_, but conformational averaging of backbone conformations is still possible on the microsecond to millisecond timescale. Due to the lack of secondary structure in the intrinsically unstructured N terminus as well as the repetitiveness of the amino acid sequence in the octarepeats, the signal overlap is so severe that sequence-specific resonance assignment for the solid-state NMR spectra was not possible.

**Figure 2:**
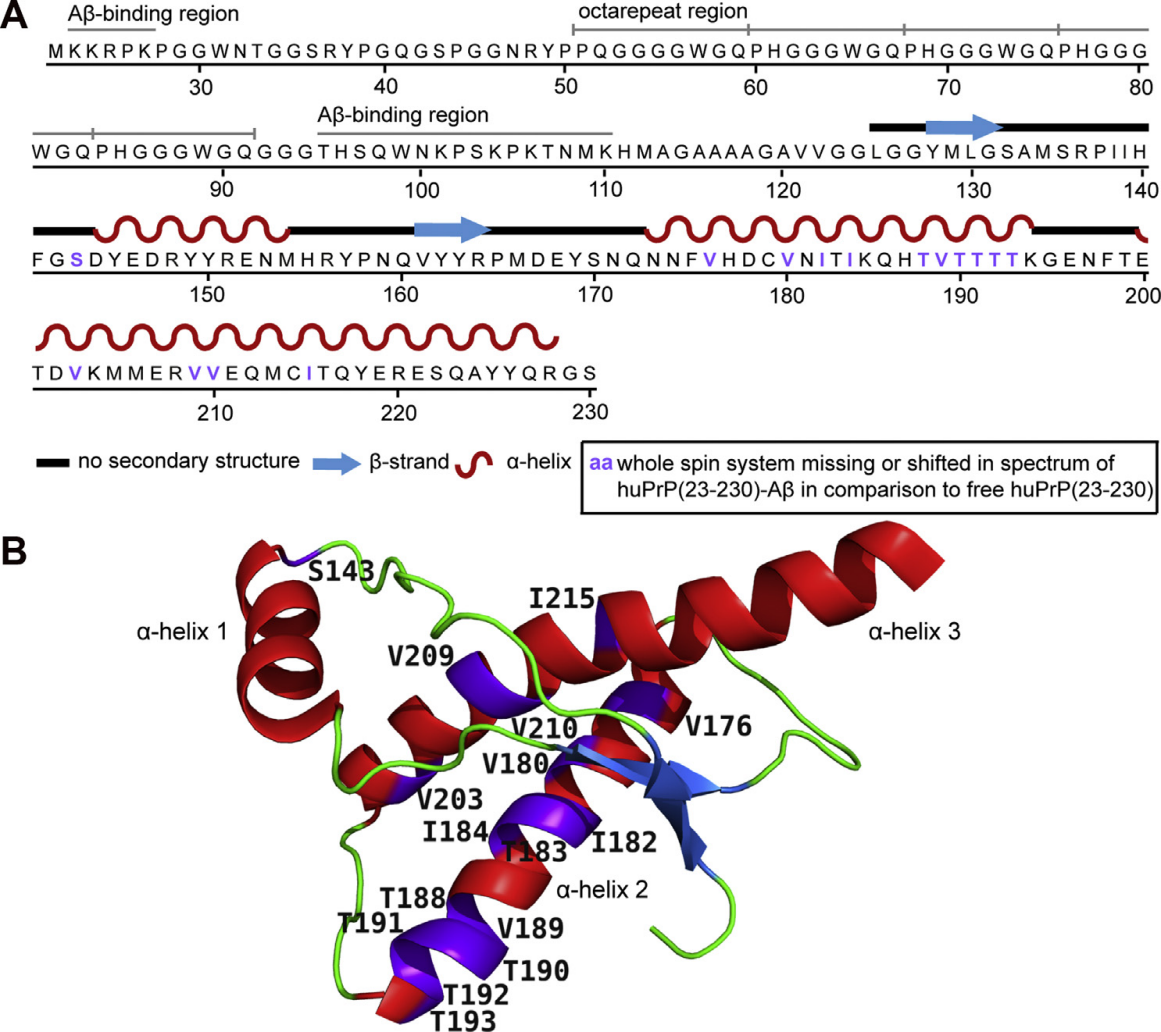
***A*, amino acid sequence of huPrP(23–230)** (48) **used in this study.** The Aβ-binding regions K23–K27 and T95–K110 (35, 36, 37, 38, 39, 40) and the five octarepeats are indicated above the sequence. *B*, 3D structure of the natively folded prion domain (residues 125–228) of full-length huPrP(23–230) in solution. β-strands are colored *blue*, α-helices *red*. Picture adapted from PDB-File 1QLZ (48). Residues whose entire spin system is missing or shifted in the PDSD spectra (Figs. S10–S12) are highlighted in *purple* in *A* and *B*.

While most of the resonances of huPrP(23–144) in complex with Aβ_oligo_ have the same chemical shifts as huPrP(23–144) in solution, some differences can be clearly seen; in particular, some Ala, Val, and Leu resonances are shifted from random coil toward α-helical secondary chemical shifts. Six out of seven Ala residues as well as both Val and Leu residues present in the sequence are located within a short stretch from residue 113 to 130, a region that starts with the so-called palindrome segment (A^113^GAAAAGA^120^) (see Fig. 2*A*). Thus, structural changes upon complexation with Aβ_oligo_ in N-terminal huPrP(23–144) seem to be confined mainly to the region between A113 and L130.

These findings are also supported by analysis of secondary chemical shifts (Fig. S8). Most secondary chemical shifts of huPrP(23–144) in solution are random coil chemical shifts indicative of a lack of regular secondary structure. Likewise, most spin systems of huPrP(23–144) in complex with Aβ_oligo_ are typical random coil chemical shifts, with some α-helical shifts found for Ala, Leu, and Val, which are not found for monomeric huPrP(23–144). Notably, almost no β-strand-like secondary chemical shifts were identified for complexed huPrP(23–144). This finding is an indication that huPrP(23–144) did not aggregate into amyloid fibrils. We also compared the chemical shifts of huPrP(23–144) fibrils (52, 53) with our correlation spectrum of huPrP(23–144)∗-Aβ (Fig. S9*A*). Most of the signals observed for fibrillar huPrP(23–144) do not overlap with the signals in our huPrP(23–144)∗-Aβ spectra. We therefore conclude that the conformations of huPrP(23–144) in huPrP(23–144)∗-Aβ and the huPrP(23–144) fibril are very different, and the interaction with Aβ_oligo_ did not induce huPrP(23–144) fibril formation.

### The C terminus of huPrP shows changes in α-helices 2 and 3 upon Aβ_oligo_ binding

**Figure 3:**
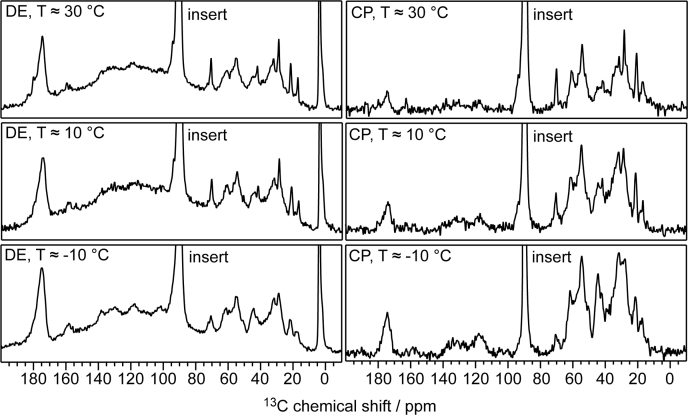
^**13**^**C Direct excitation (DE) and**^**1**^**H-**^**13**^**C CP spectra of huPrP(23–230)∗-Aβ (∗ species is**^**13**^**C,**^**15**^**N uniformly labeled) recorded at temperatures of ≈30, 10, and −10 °C and 11 kHz spinning frequency.** Recycle delays of 20 s and 2 s were used for DE and ^1^H-^13^C CP experiments, respectively. The signal at 90 ppm is caused by the rotor insert (Delrin) and is cut off for clarity. The signal at around 0 ppm in the ^13^C DE spectrum belongs to a silicone-based rotor inlet and is likewise cut off for clarity, the broad bump centred at 120 ppm however is the Teflon background of the probe. Both signals are not detected in the CP spectra. Signal intensities were scaled to the number of scans for each spectrum. Even at the lowest temperature the free water in the sample was not completely frozen, as verified by ^1^H spectra (not shown).

For full-length huPrP in complex with Aβ_oligo_, huPrP(23–230)∗-Aβ (see Table 1), no signals were detected at ≈30 °C in the INEPT spectrum (not shown), which indicates that not only the N terminus, but also the C terminus of huPrP is not highly dynamic. By contrast, excitation with ^1^H-^13^C CP results in a typical ^13^C NMR spectrum expected for a protein. To test whether the full protein is visible in the spectrum or whether a substantial part of the protein is too mobile for dipolar transfer, we compared 1D spectra obtained with ^13^C direct excitation (DE) and ^1^H-^13^C CP spectra recorded at sample temperatures of ≈30, 10, and −10 °C (Fig. 3). At all three temperatures, no substantial differences between the spectra are visible. Signal intensities in both types of spectra are roughly proportional to 1/T following Curie’s law, and at all temperatures signal intensities in CP spectra are up to two times higher than in the respective DE spectra. This is an indication that the complete huPrP molecule is fully immobilized over the whole temperature range and does not undergo major mobility changes (44). Some signals (*e.g.*, ≈70 ppm, C_β_ of Thr) show broader linewidths at lower temperatures, indicating reduced motional averaging of chemical shifts.

**Table 1:** Details of the samples used for the solid-state NMR measurements (∗ species is ^13^C, ^15^N uniformly labeled)

Sample	^13^C, ^15^N labeled species	Monomer stoichiometry Aβ:huPrP (initial mixture)	Monomer stoichiometry Aβ:huPrP (in complexes)
huPrP(23–144)∗-Aβ	huPrP(23–144)	6:1	not estimated
huPrP(23–230)∗-Aβ	huPrP(23–230)	4:1	not estimated
huPrP(23–144)-Aβ∗	Aβ(1–42)	8:1	8.6:1
huPrP(23–144)_exc_-Aβ∗	Aβ(1–42)	2:1	(3.7 ± 0.12):1

**Figure 4:**
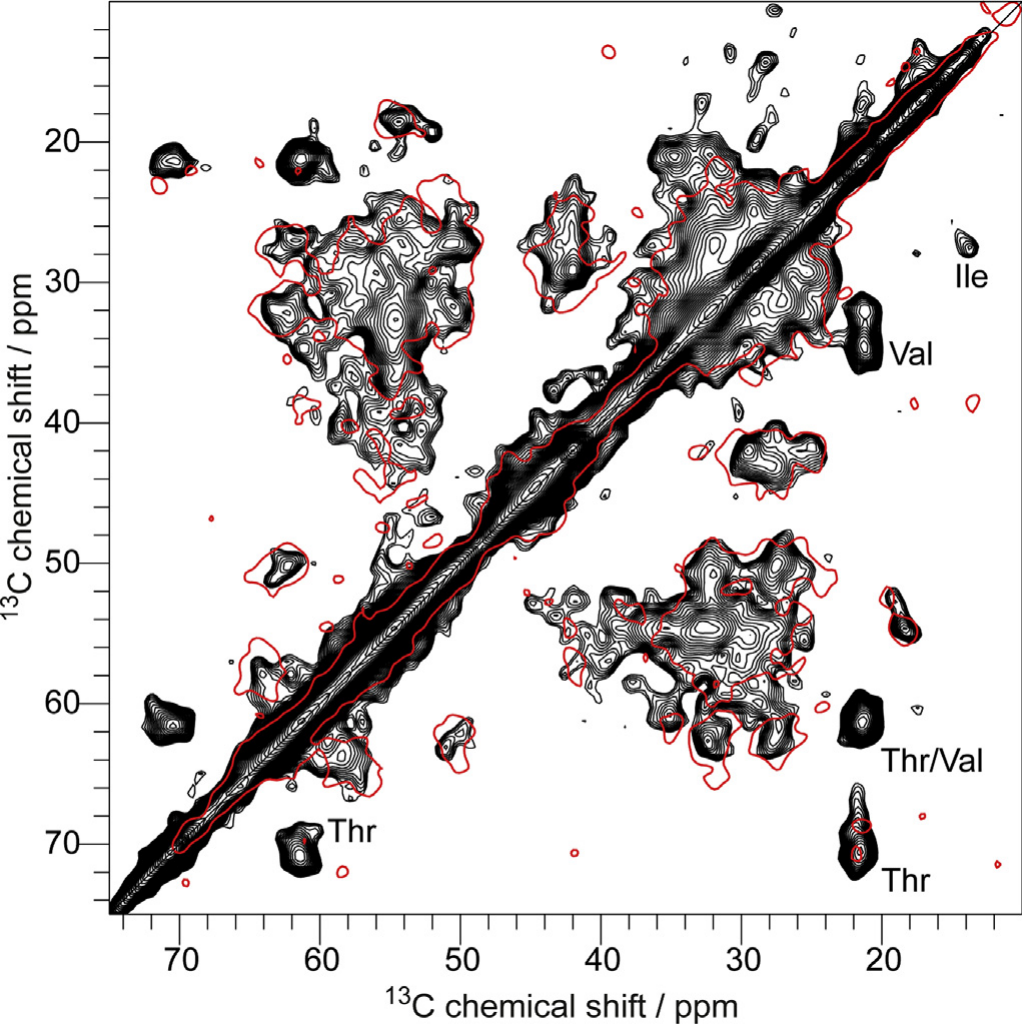
**Comparison of two PDSD spectra of huPrP(23–230)∗-Aβ (∗ species is**^**13**^**C,**^**15**^**N uniformly labeled), shown in *black*, and huPrP(23–144)∗-Aβ, shown as *red contour*.** Both spectra were recorded at a spinning frequency of 11 kHz and a mixing time of 30 ms, but the *black one* at a temperature of ≈0 °C and the *red one* at ≈−6 °C.

In Figure 4, a typical 2D PDSD spectrum of the huPrP(23–230)∗-Aβ complex is displayed. A line width of ≈1 ppm is observed for the ^13^C resonances, and due to the large number of resonances and the limited signal dispersion, the signal overlap is so substantial that a sequential resonance assignment or even a quantitative analysis of residue-specific correlations was not possible. Nevertheless, a comparison with the corresponding 2D ^13^C-^13^C correlation spectrum of the N-terminal construct huPrP(23–144) in complex with oligomeric Aβ (red outline in Fig. 4) allows some conclusions about the structure of full-length complexed huPrP: First, almost all resonances observed in the spectrum of C-terminally truncated huPrP(23–144) appear to be also visible in the spectrum of full-length huPrP(23–230)∗-Aβ (Fig. 4). These findings suggest that the C terminus of full-length huPrP(23–230) does not have a major impact on the conformation of the N terminus and its interaction with Aβ_oligo_, in line with previous results (35, 36, 37, 40). Second, the spectrum of full-length huPrP complexed by Aβ_oligo_ displays additional resonances, which are absent in the spectrum of N-terminal huPrP(23–144) in complex with Aβ_oligo_. Some of the amino acid residues occurring mainly in the C terminus (*e.g.*, Ile, Thr, and Val) give rise to cross peaks that can be unambiguously identified in 2D ^13^C-^13^C correlation spectra. However, for most C-terminal amino acid residues (*e.g.*, Asp, Glu, Tyr, etc., Fig. 2*A*), the 2D correlations overlap with other resonances and can therefore not be unambiguously assigned.

We compared our 2D ^13^C-^13^C correlation spectrum with the expected correlations between the chemical shifts obtained experimentally for natively folded full-length huPrP in solution at pH 4.5 (48). While the predicted N-terminal cross peaks (residues 23–124) superimpose well with the spectrum of huPrP(23–230)∗-Aβ, some discrepancies between the experimental and the predicted spectrum are observed for the C terminus (residues 125–230) (Fig. S10).

In particular, correlation signals for Ile, Thr, and Val in α-helical conformation from α-helices 2 and 3 in natively folded huPrP are completely missing in the experimental spectrum (Fig. S11). Instead, correlation signals for Thr and Val with secondary chemical shifts indicative of β-strands that are not observed in natively folded huPrP are clearly visible in the experimental spectrum (Figs. S10–S12). This suggests that at least for a substantial fraction of the huPrP molecules within the complex, some parts of a region between either V121 and I139 and/or V176 and I215 (located in α-helices 2 and 3) have undergone some structural rearrangements including β-strand formation (Fig. 2*B*). We could not see any fibril formation in huPrP(23–230) within the complexes; nevertheless, we overlaid our spectrum with predicted peaks for two recently published fibrils from huPrP and its fragment huPrP(94–178). The huPrP(94–178) fibril structure exhibits a β-strand in the palindrome region (54), which is likewise not supported by our α-helical-like Ala chemical shifts (Fig. S9*B*). However, a fibril structure recently published for full-length huPrP (55) (see Fig. S9*C*) shows a lot of similarities to our spectrum especially for Thr and Val residues, suggesting a rearrangement of the C terminus to more β-sheet-like chemical shifts.

### High β-strand content of Aβ_oligo_ in huPrP(23–144)-Aβ complexes

We also investigated the homogeneity and structural characteristics of Aβ_oligo_ using two samples containing uniformly ^13^C, ^15^N labeled Aβ_oligo_ in complex with nonlabeled huPrP(23–144) in two different molar ratios (Table 1). In the first sample (indicated as huPrP(23–144)-Aβ∗), the molar ratio between Aβ monomers and huPrP was roughly 8:1, whereas in the second preparation (indicated as huPrP(23–144)_exc_-Aβ∗), addition of huPrP(23–144) in excess to the Aβ oligomers resulted in a molar ratio of ≈4:1.

**Figure 5:**
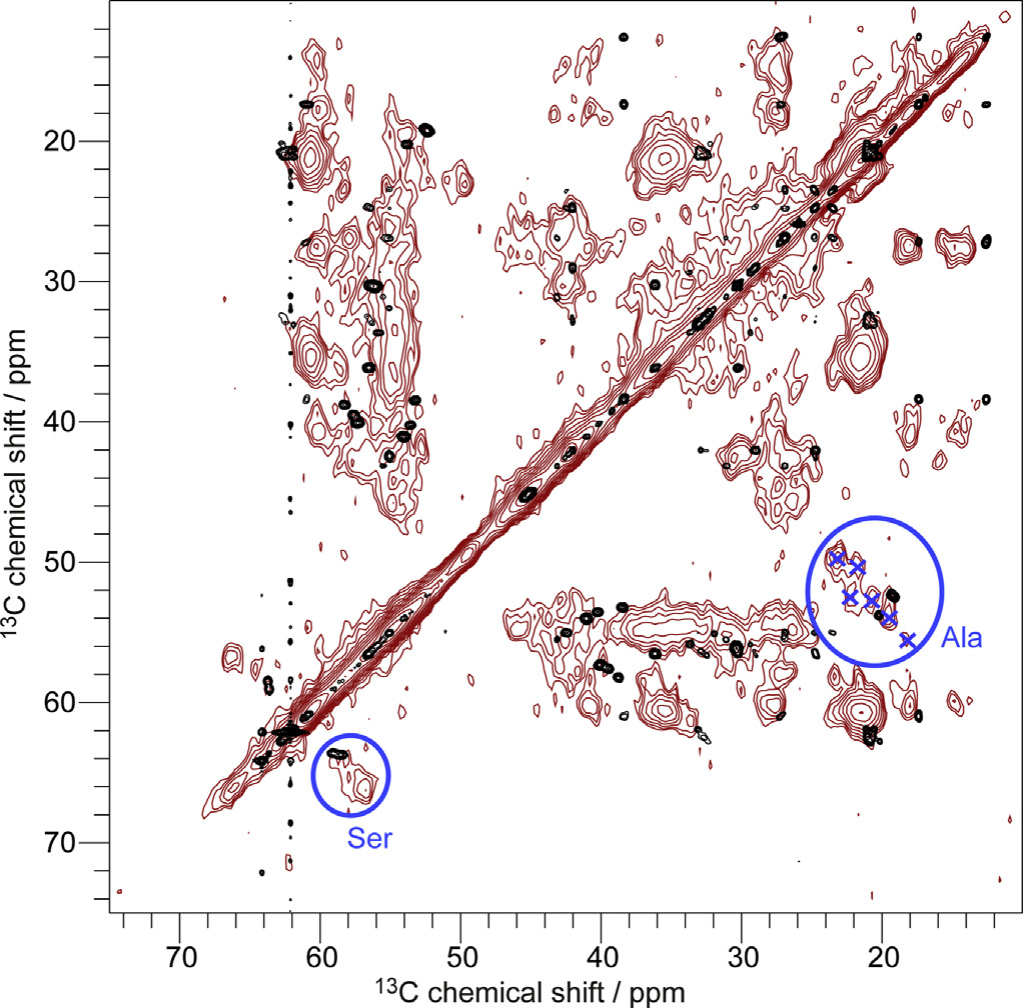
**Overlay of a PDSD spectrum of huPrP(23–144)-Aβ∗ (∗ species is**^**13**^**C,**^**15**^**N uniformly labeled), measured at a temperature of ≈0 °C, a spinning frequency of 11 kHz and a mixing time of 50 ms, in *red* with a**^**13**^**C-**^**13**^**C TOCSY spectrum of uniformly**^**13**^**C,**^**15**^**N isotope labeled Aβ monomers in solution (measured at a temperature of 5.0 °C and pH 7.2 in 30 mM Tris-HCl buffer) in *black* (the strong resonances at 62.1 ppm and 64.2 ppm with the t**_**1**_**noise are from the Tris buffer).** Ala and Ser C_α_-C_β_ peaks are highlighted with *blue circles* and six identified Ala spin systems are shown with *blue crosses*. Chemical shift differences for both residue types between the Aβ monomers (typical random coil chemical shifts) and huPrP(23–144)-Aβ∗ (β-strand-like conformations) can be observed.

INEPT spectra recorded at ≈20 °C of both samples are devoid of protein signals (not shown), whereas ^1^H-^13^C and ^1^H-^15^N CP spectra recorded at ≈0 °C display strong signals typical for all amino acid residue types (Fig. S13). These findings indicate that also the Aβ molecules are rigid in the complex. In all 2D and 3D homonuclear ^13^C-^13^C and heteronuclear ^15^N-^13^C correlation spectra (see Fig. 5 and Figs. S14–S18), linewidths are rather broad (0.9 ppm for ^13^C and 3.3 ppm for ^15^N), which is an indication for conformational heterogeneity of the Aβ molecules within the complex. In 2D ^13^C-^13^C correlation spectra (Fig. 5 and Fig. S14), ^13^C side chain and backbone resonances can be identified for almost every amino acid residue type in the sequence. For several amino acid residue types, the number of distinct spin systems visible in the spectra is larger than the number of amino acid residues of this type in the amino acid sequence. For example, six Ala spin systems have been found although the sequence of Aβ(1–42) only contains four Ala residues (Fig. 5). This means that not all Aβ molecules within the complex experience identical environments.

A comparison between a solid-state NMR ^13^C-^13^C correlation spectrum of Aβ_oligo_ in complex with huPrP(23–144) and a ^13^C-^13^C TOCSY correlation spectrum of Aβ monomers in solution (Fig. 5) reveals strong chemical shift differences and thus indicates that the Aβ monomer building blocks in oligomers have undergone significant structural changes upon oligomerization. While all signals of the solution spectrum have chemical shifts indicative of a random coil, a strong shift to chemical shifts indicative of β-strand-like secondary structure is observed for almost all spin systems of Aβ_oligo_ in the spectrum of the complex. For Cα/Cβ cross peaks of Ala, Ile, Ser, and Val (Fig. 5) in α-helical, unstructured, and β-strand-like conformations, a quantification was possible by integration of the peak regions (see Fig. S19). Hence, these residues are predominantly in a β-strand conformation. For Gly, which is a β-strand breaker, CO/Cα cross peaks are mainly indicative of random coil conformation.

Due to conformational heterogeneity, inhomogeneous line broadening, and substantial resonance overlap in the ^13^C-^13^C and ^15^N-^13^C spectra, a full sequential resonance assignment for Aβ_oligo_ in complex with huPrP was not possible. However, from a series of PDSD spectra with different mixing times as well as 2D and 3D NCACX and NCOCX spectra, it was possible to identify some interresidual correlations and to obtain site-specific assignments for some parts of Aβ in one predominant conformation (Table S1). However, it is not clear whether all assigned resonances belong to one type of conformer or to different conformers.

To elucidate whether the stoichiometry of Aβ and huPrP in the heteroassemblies has an influence on the conformations of Aβ molecules, we prepared and investigated a second sample, in which huPrP(23–144) was added in excess to ^13^C, ^15^N labeled Aβ_oligo_. In this sample, all potential huPrP-binding sites on Aβ_oligo_ should be occupied. Overall there is not much difference between sample huPrP(23–144)-Aβ∗ and huPrP(23–144)_exc_-Aβ∗ in a PDSD spectrum with a mixing time of 50 ms, except for minor changes (Fig. S20). As there are no major structural changes upon altering the huPrP concentration, we conclude that the conformational heterogeneity is not due to unoccupied huPrP-binding sites in Aβ_oligo_, but rather Aβ_oligos_ in complex with huPrP consist of inequivalent conformers and/or Aβ_oligo_ assemblies are different from each other.

## Discussion

In this study we investigated the interaction of Aβ(1–42)_oligo_ and huPrP by solid-state NMR spectroscopy. As mentioned above, Aβ(1–42)_oligo_ play a crucial role in AD, as they are neurotoxic (4). Determining structural information of Aβ(1–42)_oligo_ is challenging because of their transient and fast-aggregating nature. Therefore, trapping Aβ(1–42)_oligo_ with huPrP and inhibiting their aggregation is a convenient way to study their structure. The interaction between Aβ(1–42)_oligo_ and huPrP has also a role in AD: Nieznanski *et al.* and others showed that soluble huPrP is able to inhibit Aβ fibril formation (32, 56), particularly the naturally secreted huPrP fragment N1 (huPrP(23–111)) (33, 34) and sequesters toxic Aβ(1–42)_oligo_ (32). Additionally, soluble huPrP reduces the toxic effects of Aβ(1–42)_oligo_, as seen by us (see Fig. 1*A* and (40)) and others (32, 36).

Aside from the protective role of soluble huPrP in AD, membrane-anchored huPrP is mediating neurotoxicity of Aβ(1–42)_oligo_ (19, 24, 25) *via* Fyn-kinase (26, 27) or NMDA receptor pathways (57). Although Aβ(1–42)_oligo_ toxicity is not solely dependent on huPrP (28, 29, 30, 31), it has been shown that especially small Aβ(1–42)_oligo_ (58) and high-molecular-weight Aβ(1–42)_oligo_ (59, 60) mediate toxicity by huPrP. To target this interaction efficient inhibitors might prevent Aβ(1–42)_oligos_’ detrimental effects. Indeed, Aβ(1–42)_oligo_-binding D-enantiomeric peptides (40, 61) and antibodies (19, 62, 63, 64) have been shown to efficiently block the interaction between huPrP and Aβ(1–42)_oligo_, but more efficient inhibitors are needed. The process of research for efficient inhibitors will be speeded up by detailed knowledge of the binding between Aβ(1–42)_oligo_ and huPrP in terms of structure, because targeted research and rational design of either huPrP- or Aβ(1–42)_oligo_-binding agents will be possible.

In this study, high-molecular-weight aggregates were formed by addition of N-terminal or full-length human PrP to preformed Aβ(1–42)_oligos_. These aggregates formed immediately upon addition of huPrP, visible as the precipitation of a fine white solid powder. The rigidity of this complex was further confirmed by DE and CP NMR spectra recorded at different temperatures (see Fig. 3).

In a previous study, Kostylev *et al.* (44) investigated complexes formed between huPrP(23–111) or huPrP(23–230) and oligomeric Met-Aβ(1–42). In that study, the complexes were described as a hydrogel, and PrP molecules exhibited a higher degree of flexibility. The difference between their and our complexes may be explained by differences in the preparation of the complex (different buffer system), and in particular of the Aβ oligomers, which consisted of ≈12 molecules in the study of Kostylev *et al.* and of on average ≈23 monomers (61) in our study, which most certainly has an effect on their oligomer structure. Also, the Aβ(1–42) species used by Kostylev *et al.* contained an additional methionine residue at the N terminus, which could lead to different behavior of the Aβ(1–42) oligomers, although Silvers *et al.* (65) could show that Met-Aβ(1–42) and Aβ(1–42) fibrils exhibit the same aggregation kinetics and, except for a slight change in flexibility of the N terminus, are structurally comparable. Additionally, Kostylev *et al.* (44) used huPrP(23–111) for the majority of their investigations, whereas we used a slightly longer construct (huPrP(23–144)). This could also account for the different physical behavior in terms of flexibility.

As just mentioned, we did most of the investigations on an N-terminal construct of huPrP (huPrP(23–144)) for the following reasons: Firstly, the N terminus of huPrP is sufficient for binding Aβ(1–42)_oligo_, as shown by us (40) and others (35, 36, 37, 38, 39). Further, using huPrP(23–144) instead of huPrP(23–230) drastically reduces signal overlap in the spectra making it more straightforward to draw conclusions for the N terminus. The naturally secreted soluble N1 fragment (although slightly shorter: 23–111) exhibits a protective role in AD by reducing the cytotoxicity of Aβ(1–42)_oligo_ (34). We could show by MTT toxicity tests that also our construct huPrP(23–144) as well as soluble full-length huPrP(23–230) significantly reduced Aβ(1–42)_oligo_ toxicity (Fig. 1*A*). From a comparison of 2D ^13^C-^13^C spectra, we could show that the C terminus of huPrP(23–230) has no impact on the binding of the N terminus (23–144) to Aβ(1–42)_oligo_, suggesting that the protective effect of soluble huPrP is linked to the N terminus of huPrP. Therefore, the different roles of huPrP in the etiology of AD (*i.e.*, mediation of neurodegeneration *versus* neuroprotection) might be rather attributed to the place of action (membrane-anchored *versus* soluble) than to the length of the protein.

**Figure 6:**
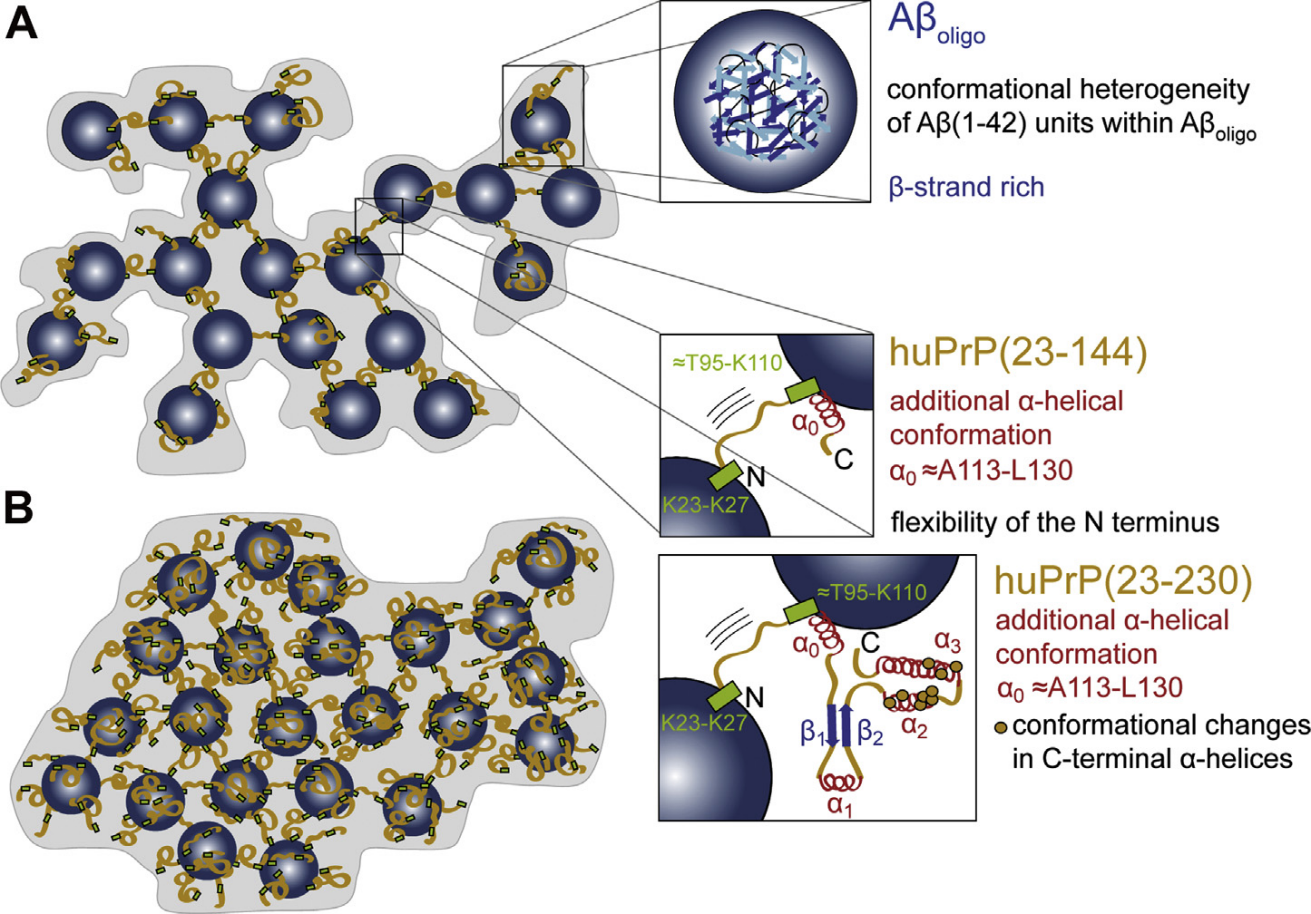
**Schematic representation showing structural features of the huPrP-Aβ**_**oligo**_**complex for *A*, low huPrP content as in the sample where huPrP(23–144) is not in excess (huPrP(23–144)-Aβ∗) and *B*, with high huPrP content as in the sample where huPrP(23–144) was added in excess (huPrP(23–144)**_**exc**_**-Aβ∗).** huPrP is shown as *orange lines*, Aβ_oligo_ as *blue spheres*, α-helices are *red*, and β-strands *blue*. Binding regions at huPrP are shown as *light green boxes*, conformational changes in the C terminus of huPrP as *orange dots*. Picture adapted from Rösener *et al.* (40).

All the findings of this study are summarized in a schematic representation of the structural features of the huPrP-Aβ_oligo_ complex in Figure 6. The N-terminal region of huPrP is rigid but has no regular secondary structure in the complex with Aβ_oligo_. This is the case for both analyzed huPrP constructs. Minor structural changes to more α-helical-like secondary structure are restricted to a region between A113 and L130, including the palindrome region AGAAAAGA. This palindrome, known as the “hydrophobic core,” is highly conserved and highly amyloidogenic (66). The palindrome segment has previously been suggested to be required for the attainment of the PrP^Sc^ conformation and to facilitate the proper association of PrP^Sc^ with PrP^C^ to enable prion propagation (67). Trapping the “hydrophobic core” by binding to Aβ(1–42)_oligo_ might explain the Aβ(1–42)-oligomer-induced inhibition of prion propagation proposed by Sarell *et al.* (68). In the already discussed above study of a hydrogel-termed complex of full-length huPrP and Aβ_oligo_ (44), the formation of two additional α-helices, one in the octarepeat region (residues 51–91) and one in the palindrome segment (A^113^GAAAAGA^120^), was postulated from the observation that chemical shifts observed for Gly and Ala are predominantly α-helical in their spectra (44). Our results support the formation of the latter α-helix in the complex with full-length huPrP. Chemical shifts of Gly residues as well as all other N-terminal residues are predominantly random coil-like in the spectra (see Fig. 1*D*), suggesting that the octarepeat region does not undergo major structural rearrangements upon complex formation. These differences are explainable by the different preparation conditions and huPrP and Aβ(1–42)_oligo_ constructs used as stated above.

For full-length huPrP in complex with Aβ_oligo_ we observed some changes for Thr and Val residues from α-helical to random coil or even β-strand-like secondary chemical shifts compared with well-folded monomeric huPrP in solution (48). The residues affected by these chemical shift changes are mainly located in α-helices 2 and 3, thus suggesting that the helical structure of this region is at least partially lost in complex with Aβ_oligo_. For huPrP in a hydrogel with Aβ_oligo_ chemical shift changes from α-helical to random coil values were also described for Thr residues, which are mainly located in α-helices 2 and 3 (44). This observation was attributed to a loss of secondary structure during liquid–liquid phase separation of PrP and in the complex with Aβ_oligo_. The loss of secondary structure in the complex with Aβ_oligo_ is confirmed by us. This observed change in secondary structure in the C-terminal domain of PrP^C^ upon binding to Aβ oligomers suggests that also the C-terminal domain of PrP^C^ interacts with Aβ_oligo_. On the contrary, the C-terminal domain is not able to bind Aβ_oligo_ on its own (40), so chemical shift changes in the C terminus might be some type of steric hindrance, a disfavor of α-helical conformations in close proximity to the β-strand-like Aβ_oligo_ or simply a structural change induced by binding of Aβ_oligo_ to the N terminus. As we could show that Aβ_oligo_ and the C-terminal fragment huPrP(121–230) do not form high-molecular-weight aggregates (40) and that this huPrP fragment does not reduce Aβ_oligo_ cytotoxicity (see Fig. 1*A*), a direct binding of Aβ_oligo_ and the C terminus of huPrP is rather unlikely. Consequently the C terminus is free to interact with any secondary (transmembrane)receptors necessary for the signal transduction, because PrP^C^ itself is no transmembrane protein and therefore requires a secondary receptor, such as NMDAR (57) or the metabotropic glutamate receptor 5 (mGluR5) (69) to facilitate Aβ_oligo_-induced neurotoxicity. Indeed the Aβ_oligo_-PrP^C^-mGluR5 complex has been shown to mediate neurotoxic Fyn-kinase pathways: Um *et al.* demonstrated that the interaction between membrane-anchored full-length PrP^C^ and mGluR5 is stabilized by Aβ_oligo_. This interaction in turn enables binding to Fyn-kinase and leads to the subsequent Fyn-kinase cascade and independent of that to increased calcium influx into the cell (69). Additionally, the Aβ_oligo_-PrP^C^-mGluR5 complex enables NMDA and muscarinic-acetylcholine receptor-independent long-term depression (70) and modulates the binding to intracellular proteins (71). It might be attractive to speculate that these interactions are mediated by a structural change in the C terminus of PrP^C^. This has to be further investigated. In another study (21), PrP constructs encompassing the N-terminal but lacking the C-terminal domain were inactive in inhibiting Aβ polymerization, even though they still bound to fibrils, whereas full-length PrP^C^ completely inhibited fibril elongation. This implied that the C-terminal domain might play some role in inhibiting polymerization. It is thus tempting to speculate that the conformational transition of the C-terminal domain to more β-strand-like structures could also be due to the incorporation into a fibril equivalent surface on Aβ oligomers. This is also supported by the finding that the C-terminal chemical shifts of huPrP overlap well with a recently published full-length huPrP fibril structure (55) (see Fig. S9*C*). Nevertheless, we should keep in mind that other studies following the aggregation of Aβ in presence of different huPrP constructs suggested the N terminus necessary for inhibiting Aβ aggregation (32, 36, 39) and also our own data argue against a direct binding of Aβ_oligo_ to the C terminus of huPrP (40), as stated above.

Aβ_oligo_ in complex with huPrP consists of nonidentical Aβ conformers. This is not surprising given the fact that the complex of huPrP(23–144) and Aβ_oligo_ contains four times more Aβ (monomer equivalent) than huPrP(23–144) molecules (40). Not every monomer within the oligomer (containing ≈23 monomer units on average (61)) might be able to bind to huPrP(23–144) in the same way and has therefore the same conformation (40), as described above. These nonidentical conformers can have different origins: (i) different types of monomers within the oligomer, because not every monomer can bind to huPrP (Aβ-huPrP *versus* Aβ-Aβ interactions); (ii) polymorphism within the oligomer independent of the binding to huPrP (iii) polymorphism between different oligomers; or (iv) a combination thereof.

**Figure 7:**
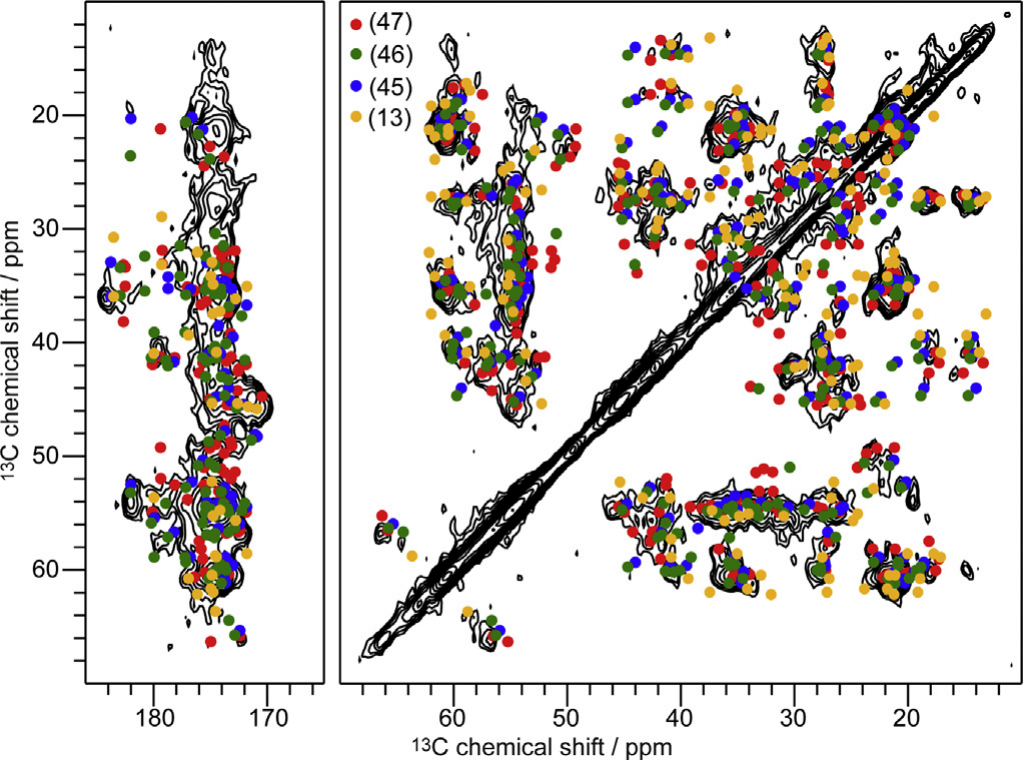
**A PDSD spectrum (measured at a temperature of ≈0 °C, a spinning frequency of 11 kHz and a mixing time of 50 ms, same spectrum as in**Fig. 5**) of huPrP(23–144)-Aβ∗ (∗ species is**^**13**^**C,**^**15**^**N uniformly labeled) in comparison with predicted cross peaks (up to two bonds) for three different fibril types, which are obtained at pH values of 2 (*red*)** (47) **or 7.4 (*green*** (46) **and *blue*** (45)**) and an artificial protofibril (*yellow*)** (13)**.** Separate overlays of this PDSD spectrum with spectra of these fibrils are shown in Figs. S21–S24.

The secondary structure of Aβ_oligo_ in complex with huPrP shows a high degree of β-strand content. Because we took care not to obtain any fibrils in our samples during preparation (see exemplarily Fig. 1*C*) and because there were no major chemical shift changes in the CP and PDSD spectra in the following 11 months (during which the sample was kept at temperatures between 4 °C and 8 °C), it is very unlikely that any significant amount of fibrils might have formed over time. Instead, the high degree of β-strand content indicates that Aβ_oligos_ already contain Aβ monomer units that have at least in part the same secondary structure as in fibrils or protofibrils, but probably differ in tertiary structure. This phenomenon has already been observed in early stage Aβ oligomers (8, 72) and is supported by the finding that huPrP-mediated toxicity depends partially on high-molecular-weight fibrillar-like Aβ_oligo_ (59, 60). Assuming that the Aβ_oligo_ preparation yielded a heterogeneous collection of fibril-like conformers in terms of secondary structure, of which most if not all were obviously elongation incompetent when trapped by adding huPrP, one would expect that the solid-state NMR resonances of Aβ_oligo_ in complex with huPrP are the sum of the resonances of different fibril conformations together with resonances from Aβ units that experience different environments due to edge effects and/or huPrP interaction. To assess the structural similarity of Aβ_oligo_ with fibrils and protofibrils, we superimposed all available resonances from three different Aβ(1–42) fibril types (45, 46, 47) and one artificial protofibril (13) with the PDSD spectrum of Aβ_oligo_ in complex with huPrP (Fig. 7 and Figs. S21–S24). A large fraction of the predicted correlations from these different protofibril and fibril types are represented by correlation peaks in our oligomer spectra, with some minor deviations found for Ala correlations. These findings suggest that the Aβ_oligo_ preparation represents a heterogeneous mixture of β-strand-rich assemblies, of which some may have the potential to evolve into the different fibril types when not trapped by huPrP. The conformational heterogeneity of Aβ_oligo_ is closely related to the polymorphism of Aβ fibrils and reflects the general propensity of Aβ to adopt variable β-structure conformers. Although we did not directly detect the binding site on Aβ_oligo_, our data suggest that the high β-strand content might be necessary for the binding, as monomers, which do not show β-strand content, have no or only little affinity for huPrP (35, 73). Aβ fibrils do bind PrP (20), but with much lower affinity than Aβ_oligo_. This might be due to the different tertiary structure compared with Aβ_oligo_. This hypothesis is also supported by others, who assume that a 3D structure rather than a special part of the sequence is necessary for binding, as elucidated by epitope mapping (37).

The propensity of huPrP to efficiently bind to Aβ_oligo_ and to “freeze” them in a nondynamic and nonelongating state allowed us to investigate the conformers of Aβ_oligo_ and the huPrP moiety by NMR over several months without noticeable changes in the sample. It is tempting to speculate whether this property of huPrP is a coincidence, or whether it is part of the long-sought function of PrP. Regardless of whether PrP inhibits elongation of Aβ oligomers and fibrils or whether PrP is a mediator of cytotoxicity of Aβ_oligos_, substances that compete with PrP for Aβ_oligo_ binding and which thus can do the same job without the potential of mediating cytotoxicity may be of high therapeutic potential.

## Experimental procedures

### Proteins

#### Aβ

For preparation of NMR samples with unlabeled Aβ, synthetic Aβ(1–42) obtained from Bachem AG was used. (For preparation of stocks see below.) Uniformly ^13^C, ^15^N labeled Aβ(1–42) was purchased from Isoloid GmbH.

#### huPrP

The purification of recombinant full-length huPrP(23–230) and C-terminally truncated huPrP(23–144) either unlabeled or uniformly ^13^C, ^15^N labeled, and of recombinant unlabeled huPrP(121–230) was performed as described previously (40).

### Preparation of Aβ(1–42) stocks

Synthetic unlabeled Aβ(1–42) (Bachem AG, 1 mg aliquot) was incubated with 700 μl hexafluoro-2-propanol (HFIP) overnight and divided into 108 μg doses in LoBind reaction tubes (Eppendorf AG). Samples were lyophilized in a rotational vacuum concentrator system connected to a cold trap (both Martin Christ Gefriertrocknungsanlagen GmbH). The lyophilizates were stored at room temperature and protected from light.

### Preparation of high-molecular-weight heteroassemblies from amyloid β oligomers and different human prion protein constructs in different molar ratios

For sample preparation, Aβ(1–42) lyophilizates (either uniformly ^13^C, ^15^N labeled or unlabeled) were dissolved in 30 mM Tris-HCl buffer, pH 7.4, yielding Aβ(1–42) concentrations of 160–300 μM. After 2 h of incubation at 22 °C and 600 rpm shaking to obtain Aβ_oligo_, either huPrP(23–144) or huPrP(23–230) was added to yield concentrations of 40 to 80 μM within the initial mixture leading to the molar ratios mentioned in Table 1. The addition of huPrP resulted in immediate sedimentation of the complex as a powder-like precipitate (40).

After addition of 0.03% of sodium azide and incubation for 30 min, the samples were centrifuged for 2 to 5 min at 16,100*g*, and the supernatant was removed. The sediment was washed twice with up to 2 ml of 30 mM Tris-HCl buffer, 0.03% sodium azide, pH 7.4 to remove excess monomeric PrP. After removal of the supernatant, the samples were transferred into 3.2 mm MAS rotors with a Hamilton syringe and centrifuged. In total, four different samples were prepared in which either huPrP or Aβ(1–42) was uniformly ^13^C, ^15^N labeled, using different huPrP constructs and molar ratios between huPrP and Aβ(1–42) (Table 1).

### Characterization by density gradient ultracentrifugation, SDS-PAGE, and RP-HPLC

For biophysical characterization of *e.g.*, sample huPrP(23–144)-Aβ∗, sucrose density gradient ultracentrifugation (DGC) was performed. To this end, 10 μl of the sedimented but unwashed sample was diluted with 90 μl of 30 mM Tris-HCl buffer, pH 7.4 and applied on a discontinuous sucrose gradient (see (40)) and centrifuged for 3 h at 259,000*g* and 4 °C. After fractionation, each of the 14 fractions was analyzed by Tris-Tricine SDS-PAGE and RP-HPLC as previously described (40) (Fig. S4).

RP-HPLC revealed the Aβ:huPrP(23–144) stoichiometry shown in Table 1 as determined in a single measurement. Sample huPrP(23–144)_exc_-Aβ∗ was not separated by DGC but measured by RP-HPLC and revealed an Aβ:huPrP(23–144) stoichiometry of 3.7 ± 0.12 to 1 after fivefold measurement of the same sample. All stoichiometries represent monomer equivalents.

### MTT cell viability tests

Potential cell viability rescue of rat pheochromocytoma PC-12 cells (Leibniz Institute DSMZ) from Aβ(1–42)_oligo_-induced toxicity through addition of soluble huPrP(23–144), huPrP(23–230), or huPrP(121–230) in a concentration-dependent manner was measured in MTT (3-(4,5-dimethylthiazol-2-yl)-2,5-diphenyltetrazolium bromide) cell viability tests (40, 61).

PC-12 cells were cultivated in RPMI 1640 medium supplemented with 10% fetal calf serum and 5% horse serum, seeded (10,000 cells in 100 μl per well) on collagen-coated 96-well plates (Gibco, Life Technologies), and incubated in a 95% humidified atmosphere with 5% CO_2_ at 37 °C for 24 h. Then final concentrations of 1 μM Aβ(1–42)_oligo_ either in the absence or after mixing and further incubation for 30 min at 22 °C with 0.02, 0.1, or 0.5 μM (final concentrations) of the respective huPrP protein were added. In addition, the toxicity of the respective huPrP proteins alone at 0.5 μM final concentrations was also determined.

After further incubation in a 95% humidified atmosphere with 5% CO_2_ at 37 °C for 24 h, cell viability was measured using the Cell Proliferation Kit I (MTT) (Roche Applied Science) according to manufacturer's protocol. The MTT formazan product was determined by measuring the absorbance at 570 nm corrected by subtraction of the absorbance at 660 nm in a FluoroStar Optima plate reader (BMG Labtech). The arithmetic mean of five independent measurements per approach ±SD was calculated. All results were normalized to untreated cells grown in medium only.

### AFM measurements

The samples used for AFM were either Aβ(1–42)_oligo_ or Aβ(1–42)_oligo_ complexed by huPrP(23–144).

For formation of Aβ(1–42)_oligo_, monomeric Aβ(1–42) was incubated at a concentration of 80 μM in 30 mM Tris-HCl, pH 7.4 for 2.5 h at 22 °C and 600 rpm shaking. For AFM the sample was then diluted to 0.44 μM with buffer and 50 μl of the solution was transferred to freshly cleaved mica and incubated for 30 min at room temperature for mica adhesion.

Preparation of the Aβ(1–42)_oligo_-huPrP(23–144) sample was done as follows: Monomeric Aβ(1–42) at a concentration of 120 μM in 30 mM Tris-HCl, pH 7.4 was incubated for 2 h, 22 °C and 600 rpm shaking. Then huPrP(23–144) was added leading to a final concentration of 80 μM Aβ(1–42) and 40 μM huPrP(23–144) in the sample. The sample was incubated further for 30 min. The generated precipitates are cleared from possibly unbound Aβ(1–42) or huPrP(23–144) by centrifugation at 16,100*g* and 4 °C for 30 min. The pellet containing the pure Aβ(1–42)_oligo_-huPrP(23–144) precipitate was washed twice with 100 μl 30 mM Tris-HCl, pH 7.4 with centrifugation steps at 16,100*g* in between. After resuspension of the condensates in 100 μl of 30 mM Tris-HCl, pH 7.4, then 50 μl of the sample was incubated on freshly cleaved mica for 30 min.

All samples were washed three times with MilliQ water and dried in a gentle stream of N_2_. Both samples were measured in a Nanowizard 3 system (JPK Instruments AG) using intermittent contact mode with a resolution of 1024 pixels and line rates of 0.5 to 1 Hz in ambient conditions with a silicon cantilever with nominal spring constant of 26 N/m and average tip radius of 7 nm (Olympus OMCL-AC160TS). Due to the curvature and adhesion of the Aβ(1–42)_oligo_-huPrP(23–144) condensates, the imaging parameters (amplitude, setpoint, and gain) had to be adapted slightly and the cantilever had to be changed often. The height image of Aβ(1–42)_oligo_ was flattened with the JPK Data Processing software 5.0.69.

### Preparation of solution NMR samples

For the sequence-specific backbone resonance assignments, samples of 0.36 mM uniformly ^13^C, ^15^N labeled huPrP(23–144) with 50 mM sodium acetate buffer in 10% (v/v) D_2_O (pH 4.5) and 0.30 mM uniformly ^13^C, ^15^N labeled huPrP(23–144) with 50 mM HEPES buffer in 10% (v/v) D_2_O (pH 7.0) were prepared as reported previously (Rösener *et al.* (40)). ^13^C-^13^C “TOtal Correlated SpectroscopY” (TOCSY) NMR measurements in solution were performed on a sample containing 0.33 mM uniformly ^13^C, ^15^N labeled huPrP(23–144) monomers (Rösener *et al.* (40)) with 0.02% (w/v) NaN_3_ in 30 mM HEPES buffer and 10% (v/v) D_2_O (pH 6.7) and on a sample of 95 μM uniformly ^13^C, ^15^N labeled Aβ(1–42) (Isoloid GmbH) in 30 mM Tris-HCl buffer and 10% (v/v) D_2_O (pH 7.2) at a temperature of 5.0 °C.

### Solid-state NMR experiments

The solid-state NMR measurements were performed either on Varian INOVA NMR spectrometers operating at field strengths of 14.1 T (ω(^1^H)/(2π) = 600 MHz) for samples huPrP(23–144)∗-Aβ, huPrP(23–230)∗-Aβ and huPrP(23–144)-Aβ∗ or a Bruker AEON 18.8 T (ω(^1^H)/(2π) = 800 MHz) spectrometer for sample huPrP(23–144)_exc_-Aβ∗, equipped with 3.2 mm standard (Varian) or wide bore (Bruker) triple-resonance MAS probes. Therefore either 3.2 mm thick wall (25 μl, for samples huPrP(23–144)∗-Aβ and huPrP(23–230)∗-Aβ) or thin wall (36 μl, for sample huPrP(23–144)-Aβ∗) rotors from Varian (Agilent) or 3.2 mm thick wall (46.7 μl, for sample huPrP(23–144)_exc_-Aβ∗) rotors from Bruker were used. For sample huPrP(23–230)∗-Aβ an insert (signal at ≈90 ppm) was used as a precaution because at the beginning of the study it was not known if PrP in huPrP(23–230)∗-Aβ was present in its pathogenic PrP^Sc^ conformation.

Sample temperatures were indirectly determined with an accuracy of ±5 °C for each spinning speed using nickelocene as an external reference (74). Initial magnetization transfer from protons to ^13^C or ^15^N was either achieved by “insensitive nuclei enhanced by polarization transfer” (INEPT) (75) to selectively excite mobile regions *via* scalar coupling through bond magnetization transfer from ^1^H to ^13^C (at ≈20, 27, or 30 °C) or by CP (measured at ≈30, 10, 7, 0, −6, or −10 °C) *via* dipolar coupling through space transfer for rigid parts. DE experiments for sample huPrP(23–230)∗-Aβ were conducted at ≈30, 10, and −10 °C. In this temperature range, the free water in the samples was not fully frozen, as could be observed from the water signal in ^1^H spectra (not shown). Additionally, several multidimensional homo- and heteronuclear correlation experiments for the assignment were recorded. Experimental details of all spectra recorded are given in Tables S2–S6. For homonuclear ^13^C-^13^C spectra, proton-driven spin diffusion (PDSD) (76) with mixing times between 10 and 300 ms was employed. Homonuclear double quantum correlation spectra were recorded with SPC5 recoupling (77).

For site-specific assignment ^15^N-^13^C correlation spectra were recorded using SPECIFIC-CP (78) for frequency selective polarization transfer from ^15^N to either ^13^Cα or ^13^CO and subsequent DARR-mixing. 2D NCA, NCACX and 3D NCACX and NCOCX spectra were used for the sequential walk through the backbone. During all acquisition and evolution times, high-power broadband proton decoupling with SPINAL phase modulation (79) (radio frequency intensity between 71 and 91 kHz) was used. All spectra were processed with NMRPipe (80) with either squared and shifted sine bell or Gaussian window functions. The line width (FWHM) was estimated in 1D-slices (spectra processed with squared sine bell shifted by 0.35π or 0.40π) of 2D PDSD or NCACX/NCOCX spectra. ^13^C chemical shifts were externally referenced with adamantane by setting the low-frequency signal of adamantane to 31.4 ppm on the DSS reference scale. ^15^N chemical shifts were indirectly referenced *via* the ^13^C chemical shifts. All resonances were assigned in CCPN (81). Integration of Aβ peaks was done in Topspin *via* the box sum method in a PDSD spectrum of huPrP(23–144)-Aβ∗, measured at a temperature of ≈0 °C, a spinning frequency of 11 kHz, and a mixing time of 50 ms.

### Solution NMR experiments

For the sequence-specific backbone resonance assignments of uniformly ^13^C, ^15^N labeled huPrP(23–144) in solution at pH 4.5, the following experiments were recorded at 5.0 °C on a Bruker AVANCE III HD 600 MHz NMR spectrometer equipped with an inverse triple-resonance probe: 2D ^1^H-^15^N HSQC (82), 3D HNCO (83), and 3D HNCACB (84) (further experimental details are given in Table S7). Sequence-specific backbone resonance assignments at pH 7.0 were obtained from 2D ^1^H-^15^N HSQC (82), 3D HNCO (83), 3D HNCACB (84), and 3D BEST-TROSY-(H)N(COCA)NH (85) experiments recorded at 5.0 °C on a Varian VNMRS 800 MHz NMR spectrometer equipped with an inverse triple-resonance probe. Two 2D ^13^C-^13^C TOCSY spectra covering either the aliphatic (bandwidth 70 ppm) or full (bandwidth 182 ppm) spectral region with a 13.6 ms 13.9 kHz (aliphatic) or 21.1 ms 15.6 kHz (full) FLOPSY-16 isotropic mixing scheme (86) of 0.33 mM uniformly ^13^C, ^15^N labeled huPrP(23–144) at 5.0 °C was recorded on a Bruker AVANCE III HD 700 MHz NMR spectrometer equipped with an inverse triple-resonance probe. Because of the comparatively low protein concentration, a 2D ^13^C-^13^C TOCSY spectrum covering the aliphatic region (bandwidth 70 ppm) with a 15.1 ms 15.6 kHz FLOPSY-16 isotropic mixing scheme (86) of 95 μM uniformly ^13^C, ^15^N labeled Aβ(1–42) at 5.0 °C was recorded on a Bruker AVANCE III HD 800 MHz NMR spectrometer equipped with a ^13^C/^15^N observe triple-resonance probe; a total of 1536 transients was collected over the course of 3 weeks and added up to further improve the signal-to-noise ratio. All triple-resonance probes were cryogenically cooled and equipped with z axis pulsed field gradient capabilities. The sample temperature was calibrated using methanol-d_4_ (87). The ^1^H_2_O resonance was suppressed by gradient coherence selection with water flip-back (88), with quadrature detection in the indirect dimensions achieved by States-TPPI (89) and the echo–antiecho method (90, 91). All solution NMR spectra were processed with NMRPipe (80) software and analyzed with NMRViewJ (92) and CCPN (81). ^1^H chemical shifts were referenced with respect to external DSS in D_2_O, ^13^C and ^15^N chemical shifts were referenced indirectly (93). RCI (50) backbone order parameters, S_RCI_^2^, were calculated from the backbone chemical shifts using TALOS-N (94) with the default parameters.

To obtain sequence-specific backbone resonance assignments for huPrP(23–144) at different pH values ranging from 4.5 to 7.0 and at a temperature of 5.0 °C, we employed the following strategy: (i) In the first step, as many resonance assignments as possible (see above) were transferred from huPrP(23–230) to the ^1^H-^15^N HSQC spectrum of huPrP(23–144) at pH 4.5 and 20.0 °C. (ii) Next, these resonance assignments were propagated along a temperature series of ^1^H-^15^N HSQC spectra of huPrP(23–144) at pH 4.5 recorded at temperatures of 15.0 °C, 10.0 °C, and 5.0 °C. (iii) The resulting sequence-specific backbone resonance assignments at pH 4.5 and 5.0 °C were verified and completed using HNCO and HNCACB triple-resonance experiments. (iv) These resonance assignments were then propagated along a pH series of ^1^H-^15^N HSQC spectra of huPrP(23–144) recorded at pH values of 5.3, 6.0, and 7.0 at a temperature of 5.0 °C. (v) Finally, the resulting sequence-specific backbone resonance assignments at pH 7.0 and 5.0 °C were verified and completed using HNCO, HNCACB, and BEST-TROSY-(H)N(COCA)NH triple-resonance experiments (Fig. S2).

## Data availability

The assigned chemical shifts of huPrP(23–144) at pH 4.5 and pH 7.0 have been deposited with the Biological Magnetic Resonance Data Bank (BMRB) under accession codes 28115 and 28116, respectively.

## Supporting information

This article contains supporting information (13, 19, 24, 25, 26, 27, 32, 33, 34, 40, 41, 47, 48, 53, 54, 55, 75, 76, 77, 78, 95, 96, 97, 98, 99).

## Conflict of interest

The authors declare that they have no conflicts of interest with the contents of this article.

## Supporting information

Supplemenatl Figures S1–S24 and Tables S1–S7
