# Supplementary material for: Structural details of amyloid β oligomers in complex with human prion protein as revealed by solid-state MAS NMR spectroscopy
Source: J Biol Chem. 2021 Mar 3;296:100499. doi: 10.1016/j.jbc.2021.100499 (PMC8042448; doi:10.1016/j.jbc.2021.100499)
Supplement: Supplemenatl Figures S1–S24 and Tables S1–S7 [file mmc1.pdf]

Supporting Information

Structural details of amyloid beta oligomers in complex with human prion protein as revealed by solid-state MAS NMR spectroscopy

Anna S. König[1,2], Nadine S. Rösener[1,2], Lothar Gremer[1,2,3], Markus Tusche[1], Daniel Flender[2#], Elke Reinartz[2], Wolfgang Hoyer[1,2], Philipp Neudecker[1,2], Dieter Willbold[1,2,3]* and Henrike Heise[1,2]*

[1]Institute of Biological Information Processing (IBI-7: Structural Biochemistry) and JuStruct: Jülich Center for Structural Biology, Forschungszentrum Jülich, Wilhelm-Johnen-Straße, 52428 Jülich, Germany
[2]Physikalische Biologie, Heinrich-Heine-Universität Düsseldorf, Universitätsstraße 1, 40225 Düsseldorf, Germany
[3]Research Center for Molecular Mechanisms of Aging and Age-Related Diseases, Moscow Institute of Physics and Technology (State University), Dolgoprudny, Russia

[#]Present address: Institut für Mikrobiologie, Universität Greifswald, Felix-Hausdorff-Str.8, 17489 Greifswald

*To whom correspondence should be addressed: Prof. Dr. Dieter Willbold: d.willbold@fz-juelich.de; Tel.: +492461612100, and Prof. Dr. Henrike Heise: h.heise@fz-juelich.de; Tel. +492461614658

Running title: Solid-state MAS NMR of the complex of huPrP and Aβ_{oligo}

Keywords: Alzheimer disease, amyloid-beta (Aβ), prion protein, oligomer, solid state NMR, solution NMR, nuclear magnetic resonance (NMR), structural biology

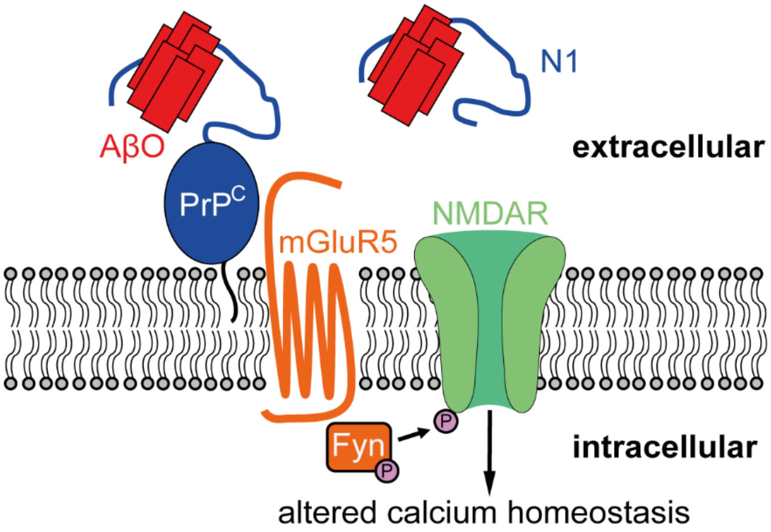

Figure S1: Concept of $A\beta_{oligo}$-binding induced signaling pathways of PrP^C resulting in neurodegeneration for full-length PrP^C or neuroprotection for N1. Specifically, $A\beta_{oligo}$ binding to membrane-anchored PrP^C mediates synapse damage (24) and the blockade of long-term potentiation by $A\beta_{oligo}$ (19,25) via activation of Fyn-kinase pathways (26,27). In contrast, soluble PrP (32) as well as its N-terminal fragment PrP(23-111) (N1) (33,34) inhibit Aβ fibrillation and sequester $A\beta_{oligos}$. Full-length PrP^C and N1 are shown in blue, $A\beta_{oligo}$ in red, the mGluR5 receptor and Fyn-kinase in orange, the NMDAR receptor in green and a phosphorylation in purple. Picture adapted from (41).

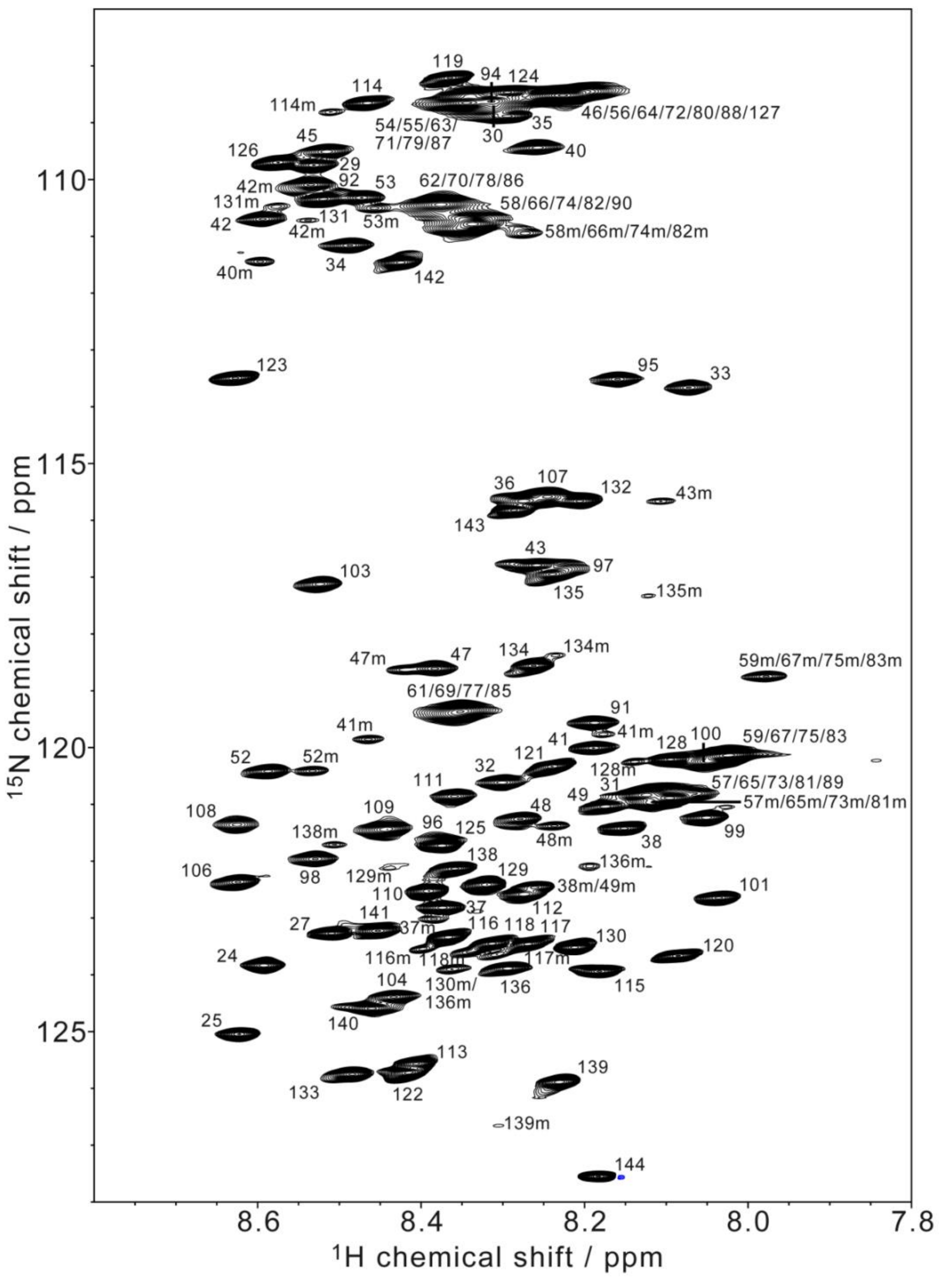

Figure S2. Backbone amide region of the ^{1}H-^{15}N HSQC spectrum of a solution of 0.30 mM [U-^{13}C,^{15}N] huPrP(23-144) in 50 mM HEPES (pH 7.0), 10 % (v/v) D_2O recorded at 5.0 °C and 800 MHz (positive contours black, negative contours blue). Backbone resonance assignments are indicated by residue numbers, minor resonances (e. g. due to proline residues in *cis* conformation or methionine oxidation) are indicated by an m. Four octarepeats (P51 to Q91) share the same sequence (**Figure 2A**) and could not be resolved. The backbone amide resonance of G93 could not be identified due to severe resonance overlap.

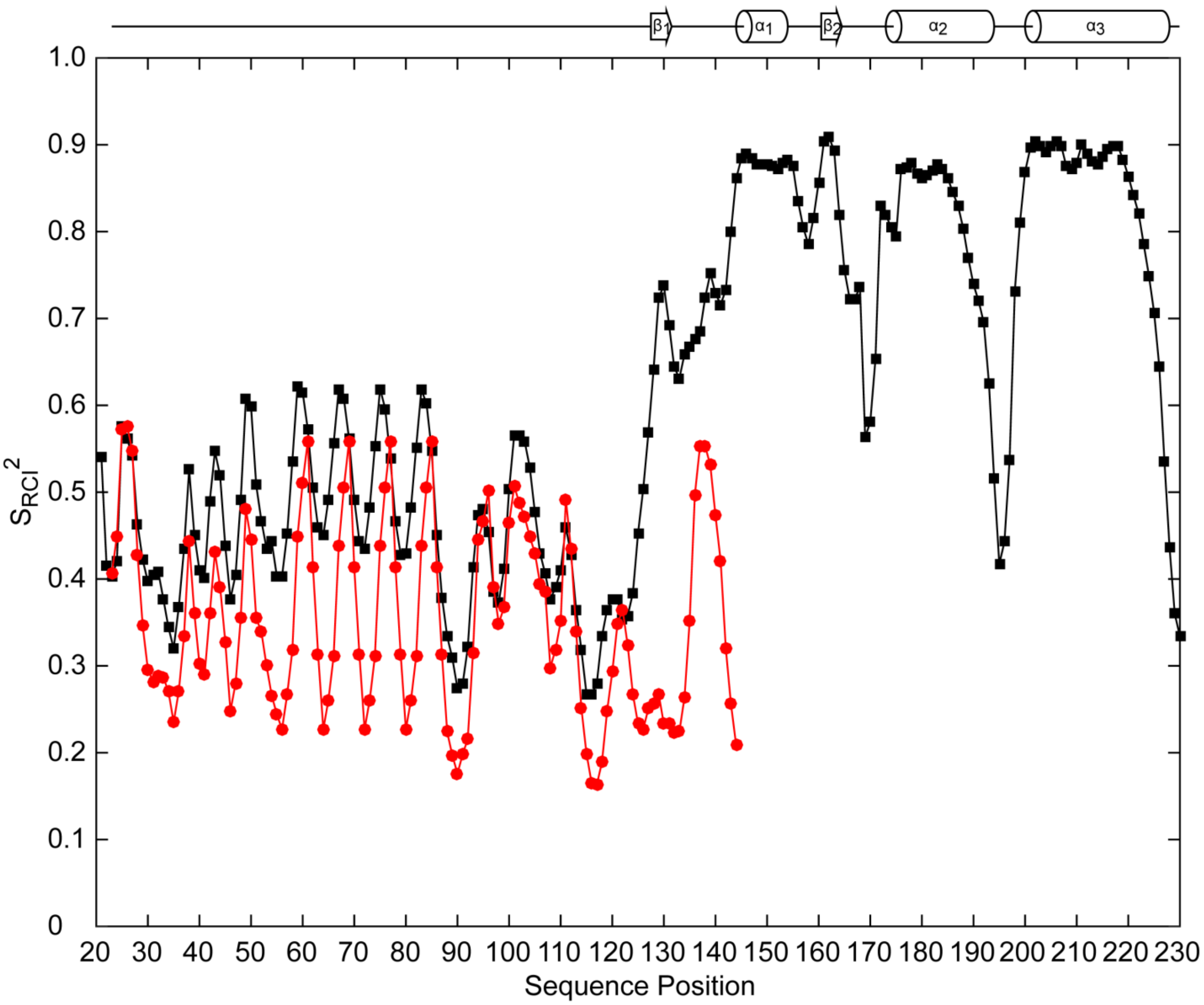

Figure S3. Backbone order parameters predicted from the Random Coil Index (RCI), S_{RCI}^2, for huPrP(23-230) at pH 4.5 (BMRB 4402, ref. (48), black) and huPrP(23-144) at pH 7.0 (BMRB 28116, this work, red), as calculated by TALOS-N (94) with the default parameters. Assigned chemical shifts for the short N-terminal cloning artifact (Gly for huPrP(23-144), Gly-Ser for huPrP(23-230)) were included. Full-length huPrP(23-230) consists of a highly disordered N-terminal region comprising residues 23 to 124 with chemical shifts very close to random coil values and concomitantly low S_{RCI}^2 values below about 0.6 (black), and a globular C-terminal prion domain comprising residues 125 to 228, whose regular secondary structure elements are indicated above the figure. Upon truncation, residues 125 to 144 of huPrP(23-144) also become disordered, with chemical shifts very close to random coil values (**Figure S2**) and concomitantly low S_{RCI}^2 values below ≈ 0.6 for all residues from 23 to 144 (red).

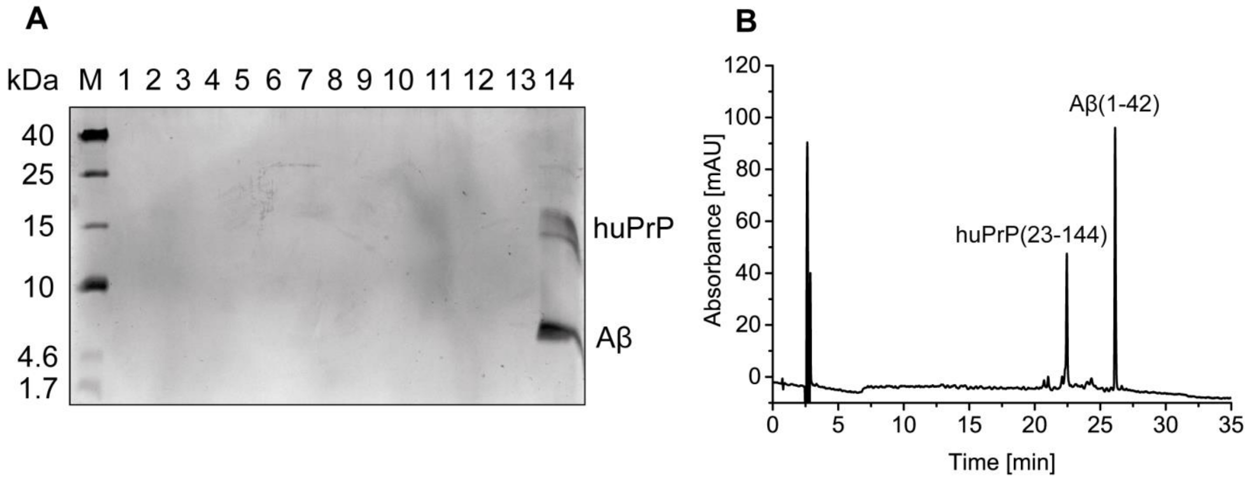

Figure S4. A. Sucrose DGC (40) of a tenfold dilution of huPrP(23-144)-Aβ* (* species is ^{13}C, ^{15}N uniformly labeled) before washing shows huPrP and Aβ only in fraction 14, corresponding to the highest density (40). **B.** Quantitative analysis by RP-HPLC on fraction 14 of the DGC revealed an Aβ(1-42) to huPrP(23-144) stoichiometry of 8.6 to 1 (monomer equivalents, single measurement).

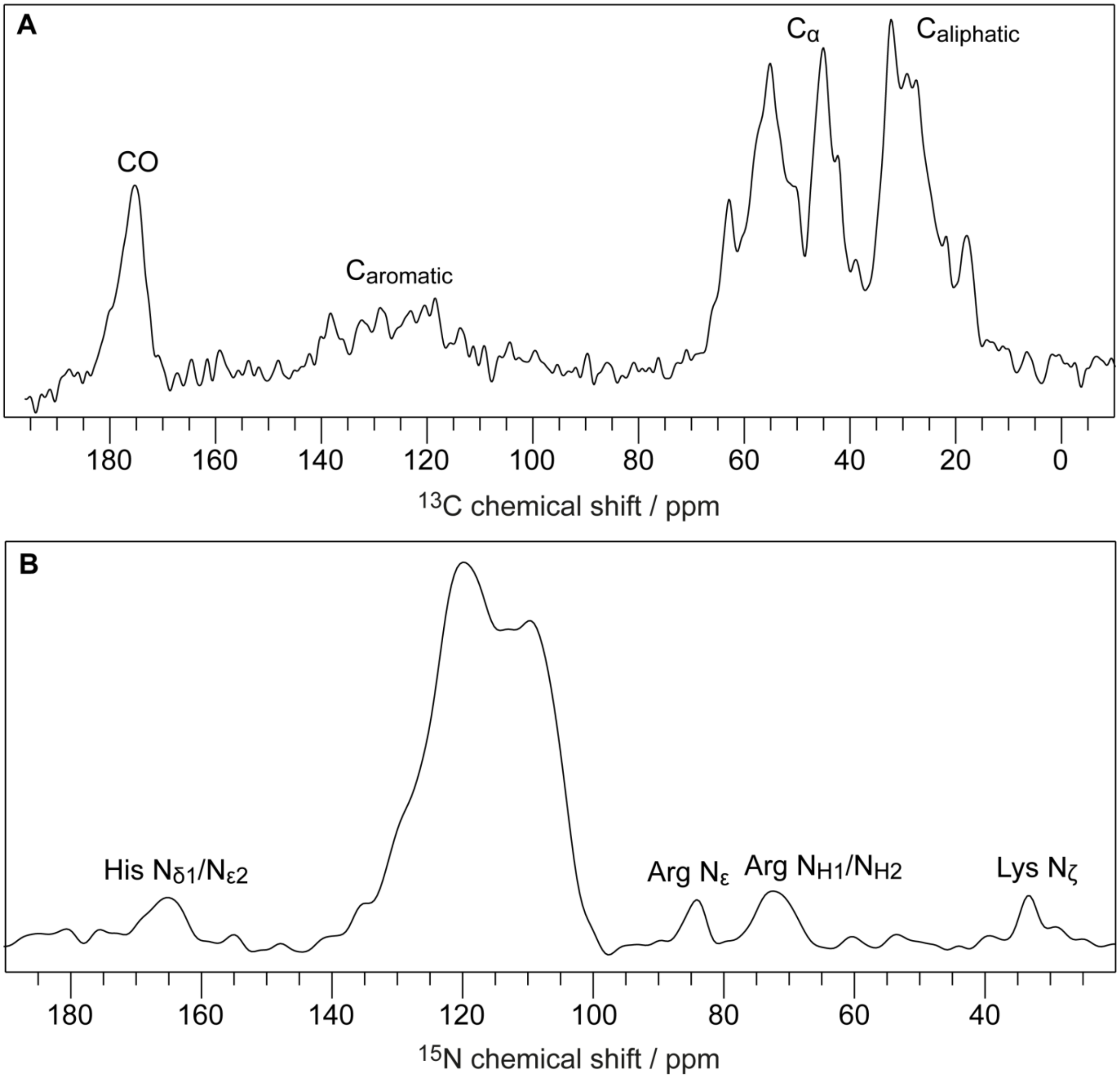

Figure S5. A. ^{1}H-^{13}C CP spectrum of huPrP(23-144)*-Aβ (* species is ^{13}C, ^{15}N uniformly labeled), recorded at a temperature of ≈ 0 °C, at a spinning frequency of 11 kHz and 256 scans. **B.** ^{1}H-^{15}N CP spectrum of huPrP(23-144)*-Aβ, measured at a temperature of ≈ -6 °C, a spinning frequency of 11 kHz and 2000 scans.

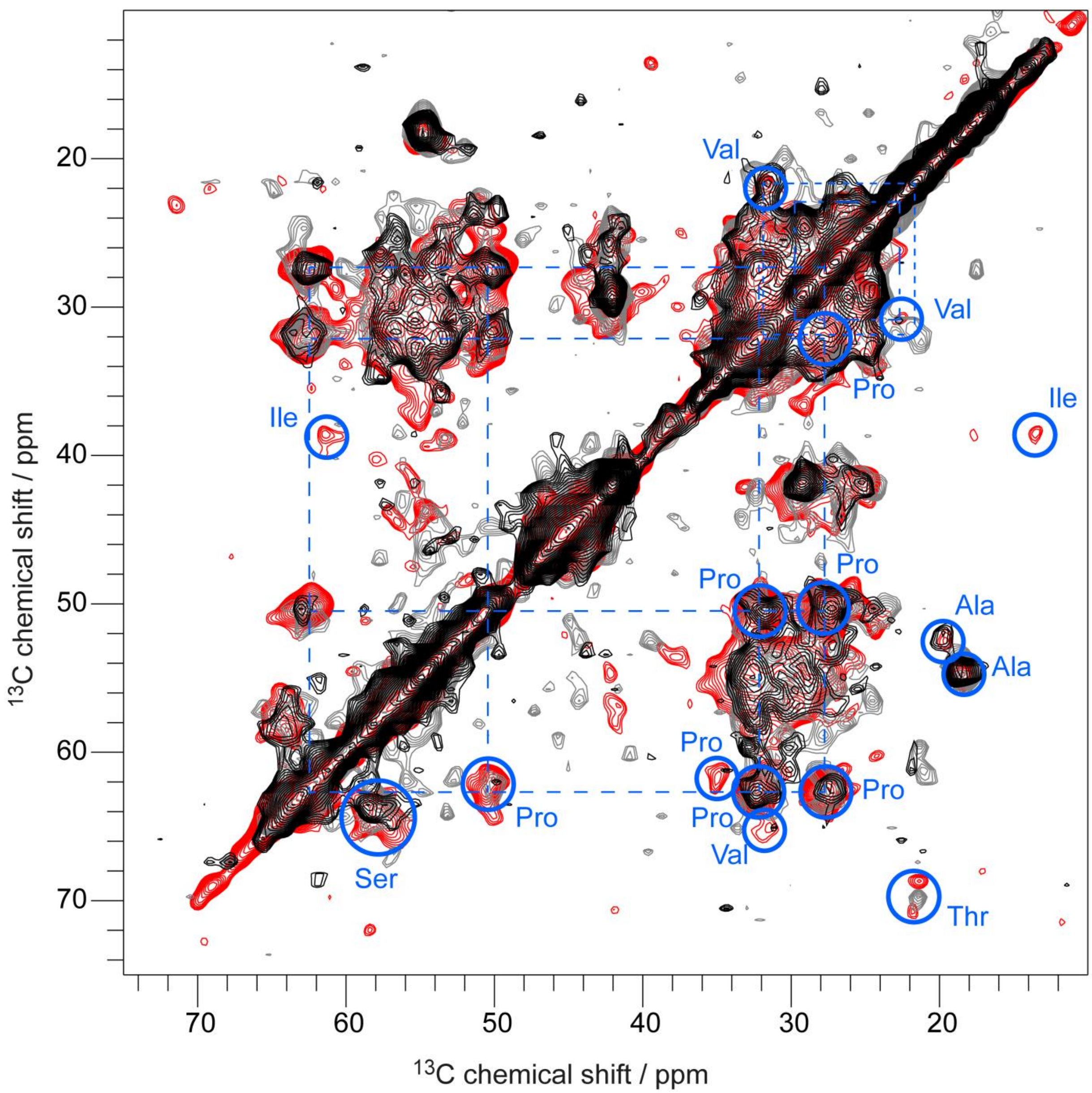

Figure S6. PDSD spectra of huPrP(23-144)*-Aβ (* species is ^{13}C, ^{15}N uniformly labeled), recorded at a spinning frequency of 11 kHz, a temperature of ≈ -6 °C and a mixing time of 30 ms (red) or a temperature of ≈ 0 °C and a mixing time of 50 ms (black) or 100 ms (grey). Blue circles indicate some identified amino acid types, dashed lines Pro and Val connections.

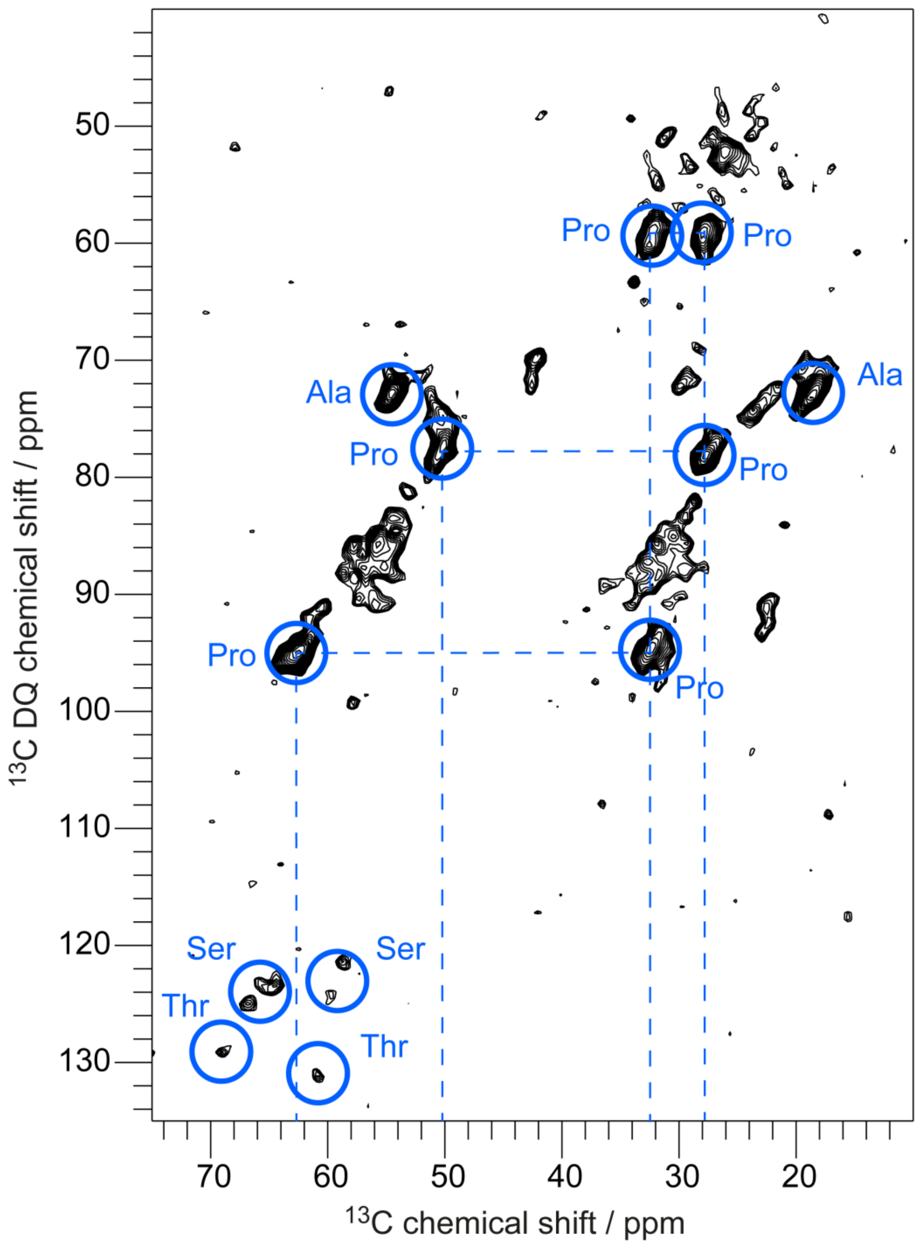

Figure S7. Double-quantum (DQ) correlation spectrum of huPrP(23-144)*-Aβ (* species is ^{13}C, ^{15}N uniformly labeled) with SPC5-recoupling, recorded at a temperature of ≈ -6 °C and a spinning frequency of 8 kHz. Blue circles indicate identified amino acid types, dashed lines Pro connections.

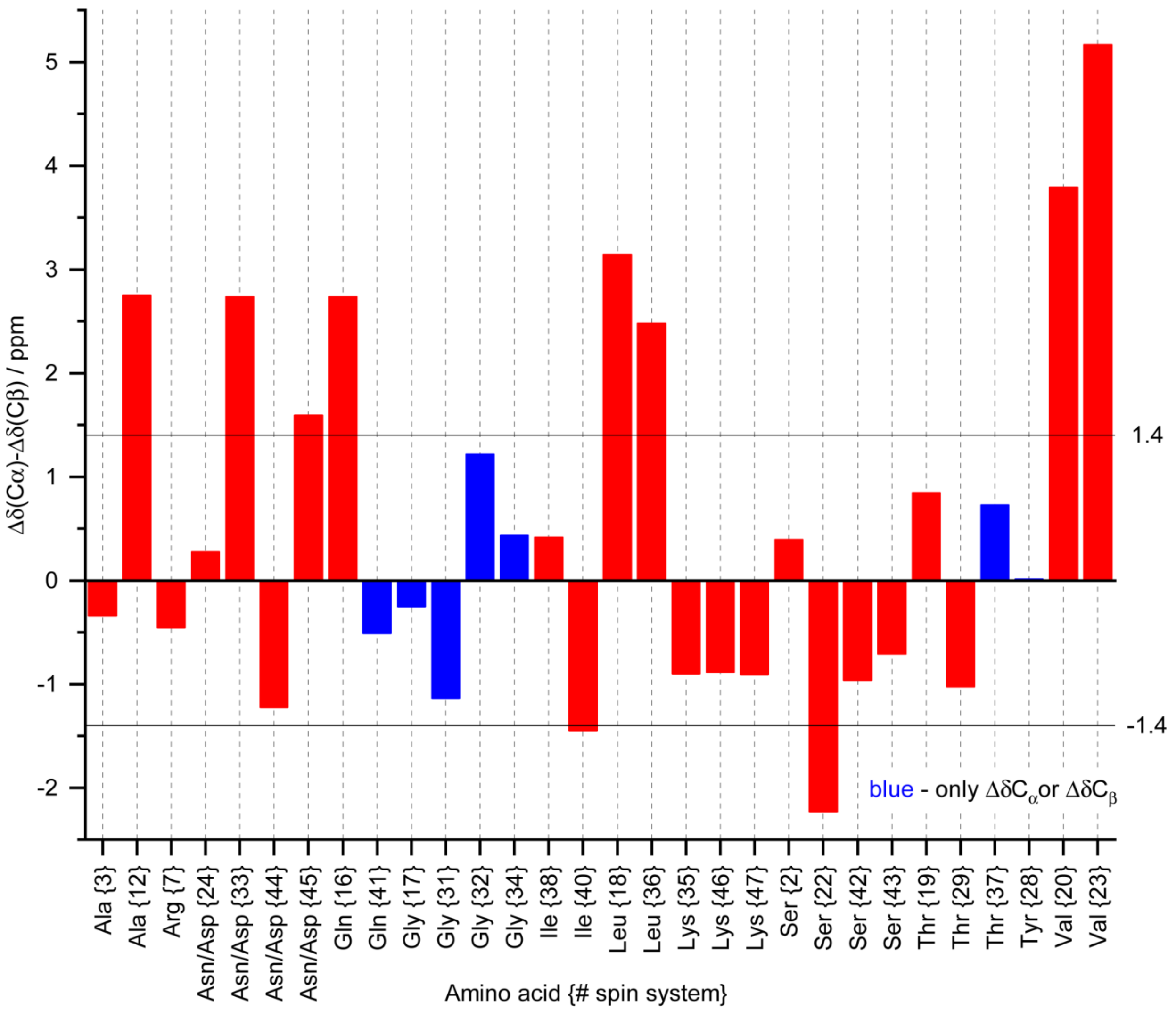

Figure S8. Secondary chemical shifts for huPrP(23-144)*-Aβ (* species is ^{13}C, ^{15}N uniformly labeled). Secondary chemical shifts are calculated as ($C\alpha_{exp}$-$C\alpha_{rc}$)-($C\beta_{exp}$-$C\beta_{rc}$), values above 1.4 ppm are indicative of α-helical-like secondary structure, values below – 1.4 ppm are indicative of β-strand-like secondary structure. In absence of a sequential assignment an arbitrary number for the spin system is given in braces. Note that blue bars indicate spin systems where only either a Cα or Cβ chemical shift was available, therefore ($C\alpha_{exp}$-$C\alpha_{rc}$) or ($C\beta_{exp}$-$C\beta_{rc}$) was calculated, respectively.

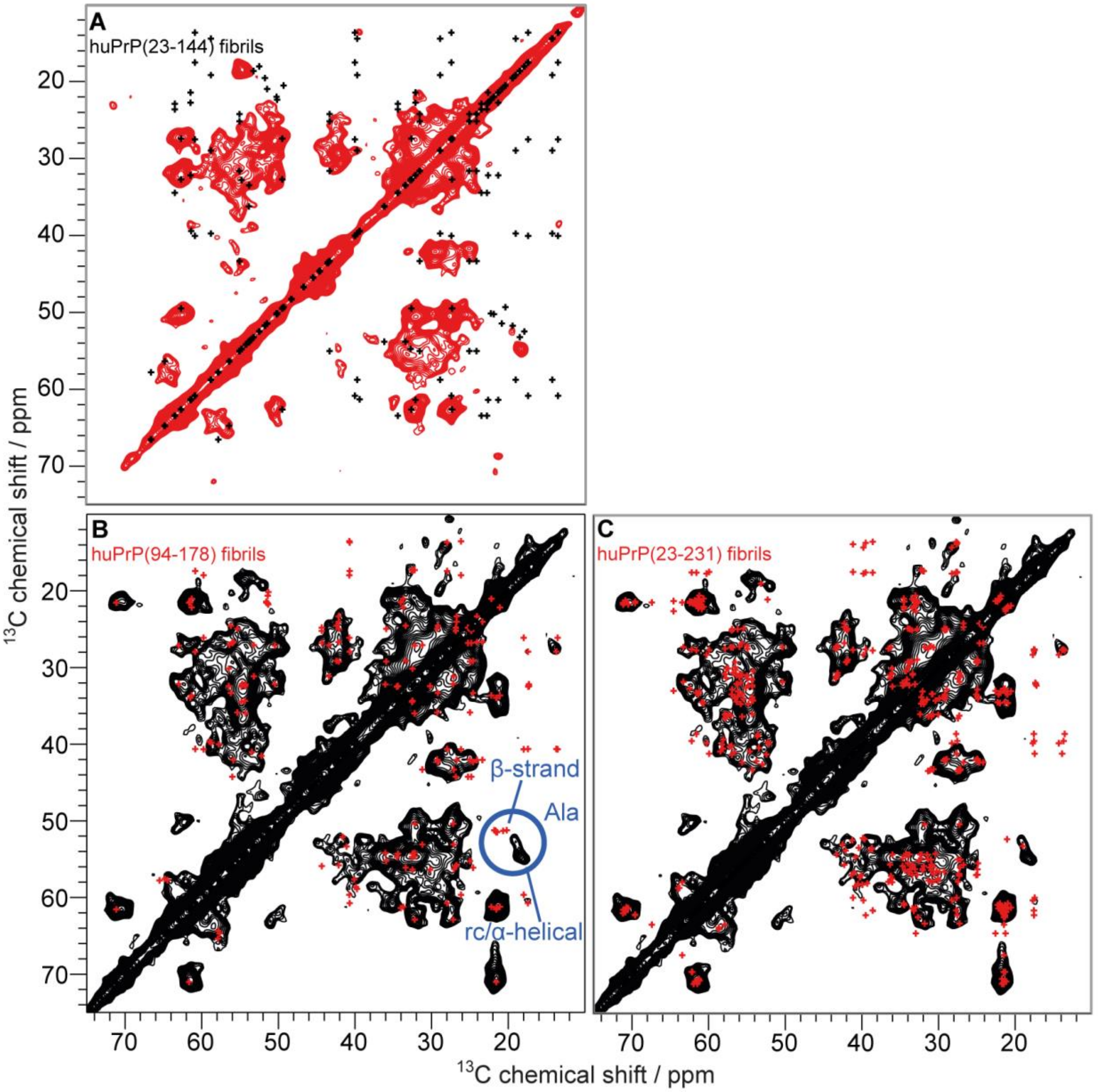

Figure S9. A. Comparison of a PDSD spectrum (recorded at a temperature of ≈ -6 °C, a spinning frequency of 11 kHz and a mixing time of 30 ms, same as in **Figure 1C**) of huPrP(23-144)*-Aβ (* species is ^{13}C, ^{15}N uniformly labeled) with the chemical shifts of a huPrP(23-144) fibril recorded by Theint et al. (BMRB entry 26925) (53), shown as black crosses. **B.** Comparison of a PDSD spectrum (black, recorded at a temperature of ≈ 0 °C, a spinning frequency of 11 kHz and a mixing time of 30 ms, same as in **Figure 4**) of huPrP(23-230)*-Aβ (* species is ^{13}C, ^{15}N uniformly labeled) with predicted chemical shifts (ShiftX2 (95)) of a huPrP(94-178) fibril structure determined through Cryo-EM by Glynn et al. (PDB 6UUR) (54), shown as red crosses. The difference in Ala chemical shifts (rc or α-helical like in this study vs. β-strand like in (54)) is highlighted in blue. **C.** Comparison of a PDSD spectrum (black, recorded at a temperature of ≈ 0 °C, a spinning frequency of 11 kHz and a mixing time of 30 ms, same as in **Figure 4**) of huPrP(23-230)*-Aβ (* species is ^{13}C, ^{15}N uniformly labeled) with predicted chemical shifts (ShiftX2 (95)) of a huPrP(23-231) fibril structure determined through Cryo-EM by Wang et al. (PDB 6LNI) (55), shown as red crosses. Values for unstructured regions are taken from the BMRB (random coil value).

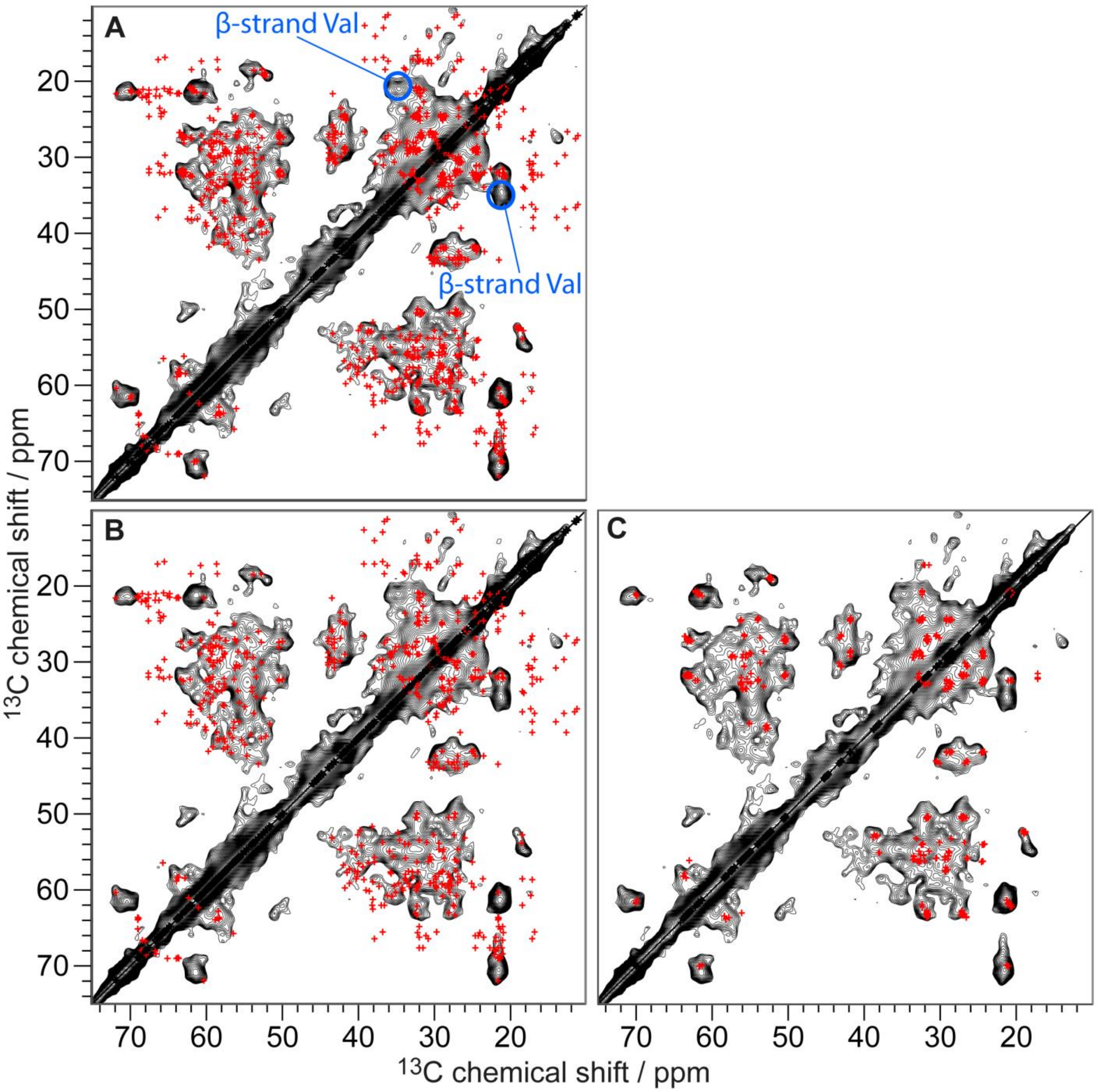

Figure S10. Comparison of a PDSD spectrum of huPrP(23-230)*-Aβ (black) (* species is ^{13}C, ^{15}N uniformly labeled) to predicted peaks, indicated as red crosses. Measured chemical shifts of Zahn et al. (BMRB entry 4402) were used for the prediction. For those carbon atoms in the structured C terminus (starting from residue 125) for which no chemical shift assignments were made by Zahn et al., chemical shifts were predicted from the PDB-structure of monomeric huPrP (PDB-Entry 1QLZ) (48) with SHIFTX2 (95). For residues from the unstructured N-terminal part for which no chemical shift assignments were made by Zahn et al., random coil BMRB values (96) were used. Cross peaks of up to two bonds were included in the prediction. Full spin-systems were simulated, but at 30 ms mixing time not all cross-correlations necessarily show up with sufficient intensity in the experimental spectrum. **A.** Values for residues MK23-S230, **B.** L125-S230 (C terminus only), and **C.** MK23-G124 (N terminus only) were used. The separation between N-terminal and C-terminal regions is made between G124 and L125, where the ordered region of the solution NMR structure starts (**Figure 2A**). The PDSD spectrum was recorded at a temperature of ≈ 0 °C, a spinning frequency of 11 kHz and a mixing time of 30 ms. β-strand-like Val is indicated with blue circles, it is the only peak in the spectrum of huPrP(23-230)*-Aβ which is not superimposed at all. Note that

these structural changes are not due to the different pH of huPrP(23-230)*-Aβ (pH 7.4) and soluble huPrP(23-230) (pH 4.5), as the conformation of the globular domain (residues 125 to 230) of monomeric huPrP(23-230) at pH 4.5 and pH 7.0 is extremely similar (97).

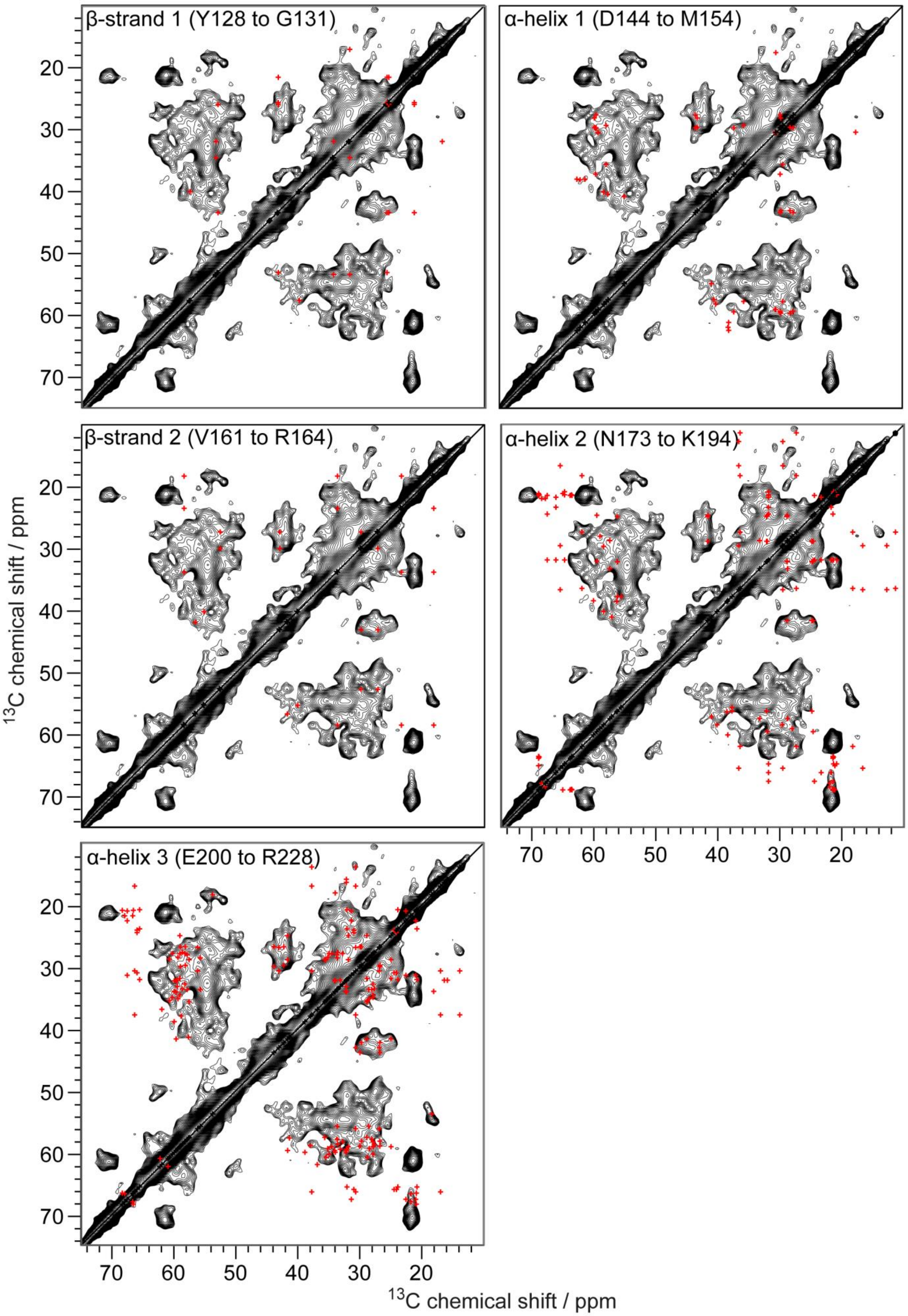
β-strand 1 (Y128 to G131)
α-helix 1 (D144 to M154)
β-strand 2 (V161 to R164)
α-helix 2 (N173 to K194)
α-helix 3 (E200 to R228)
20
30
40
50
60
70
^{13}C chemical shift / ppm
^{13}C chemical shift / ppm

Figure S11. Comparison of a PDSD spectrum of huPrP(23-230)*-Aβ (black) (* species is ^{13}C, ^{15}N uniformly labeled) to predicted peaks, indicated as red crosses. Measured chemical shifts of Zahn et al. (BMRB entry 4402) were used for the prediction. For those carbon atoms in the structured C terminus (starting from residue 125) for which no chemical shift assignments were made by Zahn et al., chemical shifts were predicted from the PDB-structure of monomeric huPrP(23-230) (PDB-Entry 1QLZ) (48) with SHIFTX2 (95). Cross peaks of up to two bonds were included in the prediction. Full spin-systems were simulated, but at 30 ms mixing time not all cross-correlations necessarily show up with sufficient intensity in the experimental spectrum. (β-strand 1) Values for residues Y128 to G131, (α-helix 1) D144 to M154, (β-strand 2) V161 to R164, (α-helix 2) N173 to K194 and (α-helix 3) E200 to R228 were used. The PDSD spectrum was recorded at a temperature of ≈ 0 °C, a spinning frequency of 11 kHz and a mixing time of 30 ms. Except for longer correlations over three or four bonds, some side chains of Leu, Met and V161, the resonances of the two β-strands and the first α-helix determined by Zahn et al. align well with the resonances of huPrP(23-230)*-Aβ. But as these resonances determined by Zahn et al. overlap with other resonances in the spectrum of huPrP(23-230)*-Aβ, it is possible that they are either not visible or shifted. Therefore, no conclusion can be drawn about the conservation of the two β-strands and the first α-helix. For the last two α-helices also longer correlations are missing. But interestingly all α-helical like Ile, Val and, in the second α-helix, Thr resonances are missing, too. This means Ile, Val and Thr are more random coil or β-strand like in huPrP(23-230)*-Aβ or partly undetectable, i.e. flexible on an intermediate time scale (as no signals are observed in an INEPT experiment (data not shown)).

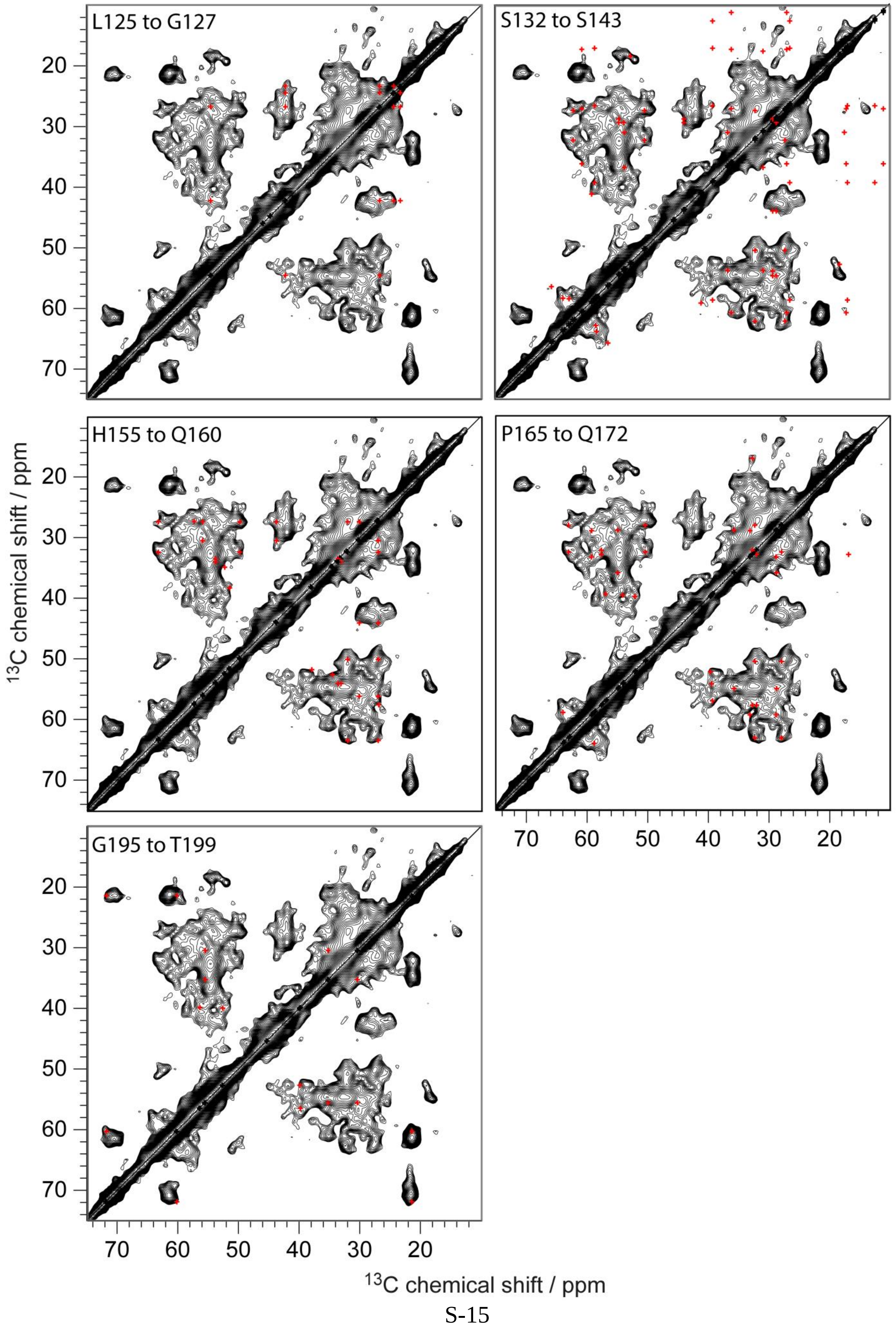

L125 to G127
S132 to S143
H155 to Q160
P165 to Q172
G195 to T199
^{13}C chemical shift / ppm
^{13}C chemical shift / ppm

Figure S12. Comparison of a PDSD spectrum of huPrP(23-230)*-Aβ (black) (* species is ^{13}C, ^{15}N uniformly labeled) to predicted peaks, indicated as red crosses. Measured chemical shifts of Zahn et al. (BMRB entry 4402) were used for the prediction. For those carbon atoms in the structured C terminus (starting from residue 125) for which no chemical shift assignments were made by Zahn et al., chemical shifts were predicted from the PDB-structure of monomeric huPrP (PDB-Entry 1QLZ) (48) with SHIFTX2 (95). Cross peaks of up to two bonds were included in the prediction. Full spin-systems were simulated, but at 30 ms mixing time not all cross-correlations necessarily show up with sufficient intensity in the experimental spectrum. Here it is shown for all loop regions between the β-strands and α-helices, namely L125 to G127, S132 to S143, H155 to Q160, P165 to Q172 and G195 to T199. The PDSD spectrum was recorded at a temperature of ≈ 0 °C, a spinning frequency of 11 kHz and a mixing time of 30 ms. In the loop regions between the β-strands and α-helices only correlations over two or more bonds plus S143 are missing or shifted.

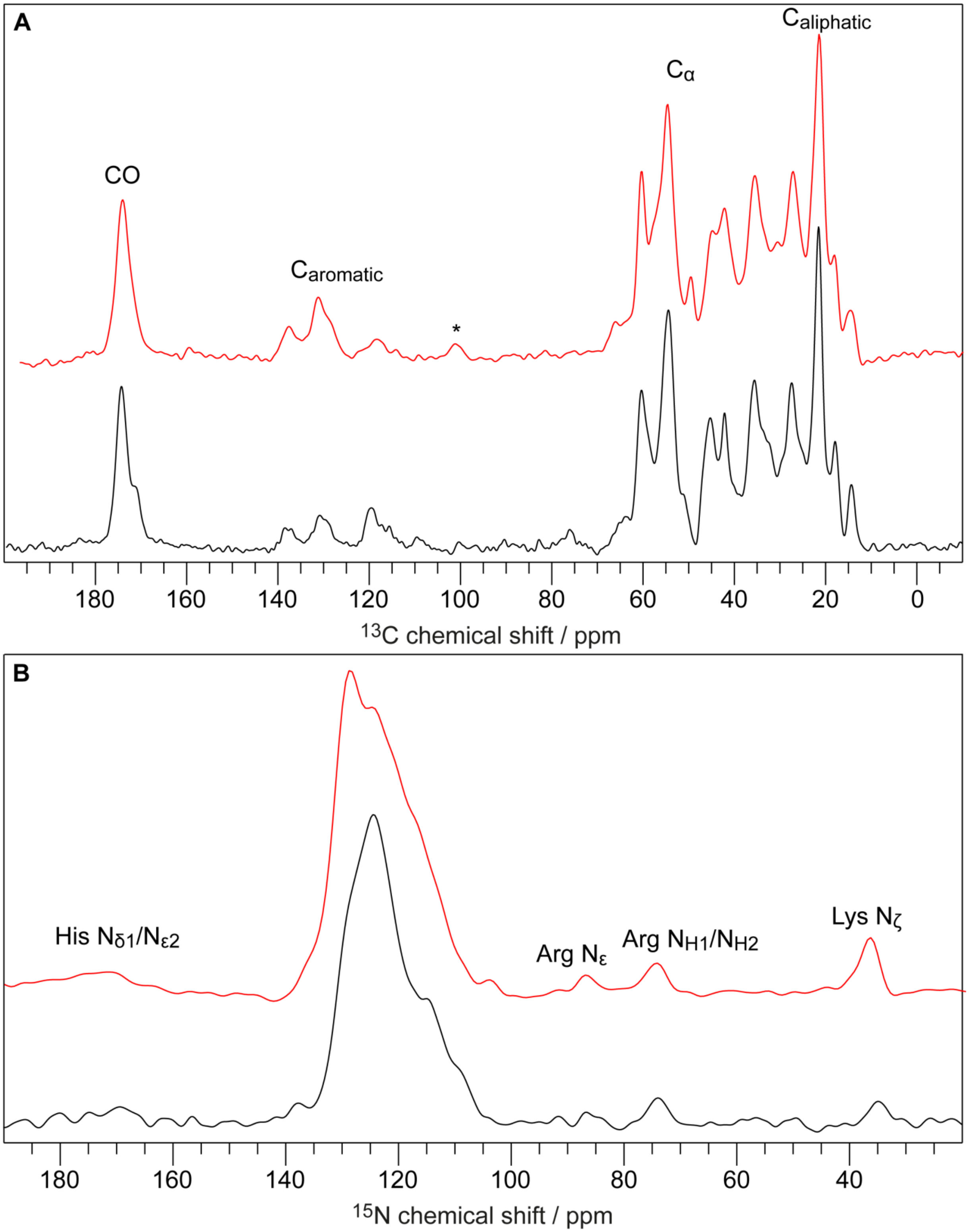

Figure S13. A. (red) ^{1}H-^{13}C CP spectrum of huPrP(23-144)-Aβ* (* species is ^{13}C, ^{15}N uniformly labeled), recorded at a temperature of ≈ 0 °C, at a spinning frequency of 11 kHz and 128 scans. Spinning side bands are marked with an asterisk. **A.** (black) ^{1}H-^{13}C CP spectrum of huPrP(23-144)$_{exc}$-Aβ* ($_{exc}$ huPrP is in excess; * species is ^{13}C, ^{15}N uniformly labeled), recorded at a temperature of ≈ 7 °C, at a spinning frequency of 5

kHz and 256 scans. **B.** (red) ^{1}H-^{15}N CP spectrum of huPrP(23-144)-Aβ*, recorded at a temperature of ≈ 0 °C, a spinning frequency of 11 kHz and 2000 scans. **B.** (black) ^{1}H-^{15}N CP spectrum of huPrP(23-144)$_{exc}$-Aβ*, recorded at a temperature of ≈ 0 °C, a spinning frequency of 11 kHz and 2048 scans.

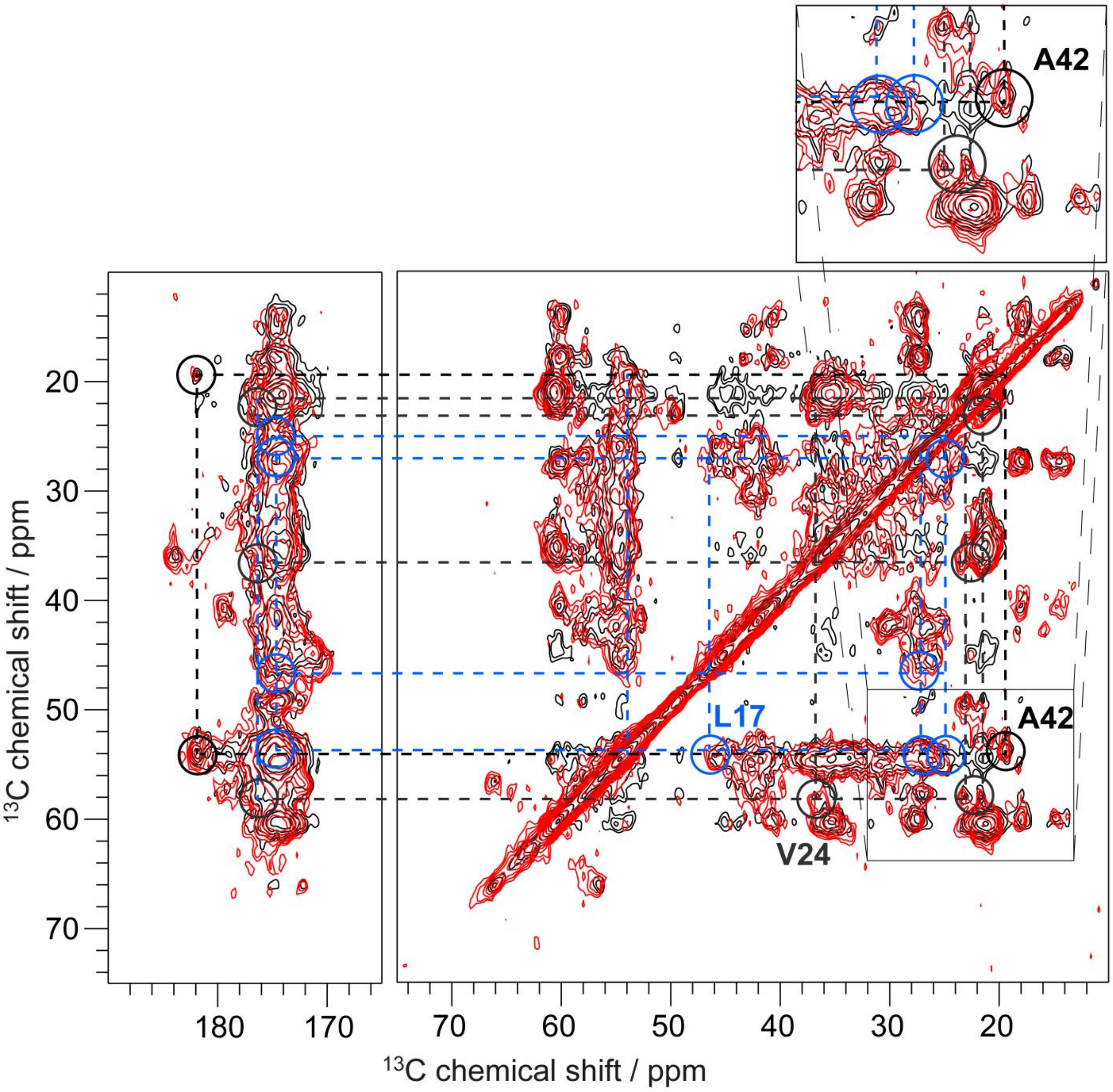

Figure S14. Two PDSD spectra of huPrP(23-144)-Aβ* (* species is ^{13}C, ^{15}N uniformly labeled), recorded at a temperature of ≈ 0 °C, a spinning frequency of 11 kHz and a mixing time of 50 ms (red) and 200 ms (black). Three identified residues are shown exemplarily with colored circles and dashed lines: L17 (blue), V24 (grey) and A42 (black). For L17 $C_{\delta1}$ and $C_{\delta2}$ chemical shifts are not separated due to the low signal dispersion. In contrast, V24 shows two separated $C_{\gamma1}$ and $C_{\gamma2}$ chemical shifts, which has been also observed in the Aβ(1-42) fibril polymorph of Gremer et al. (47). Note the high CO chemical shift of A42 (182 ppm) (left part, black circles). This can be explained by a free and deprotonated state of the C-terminal carboxyl group. At 200 ms mixing time several multi-bond and inter-residual correlations appear, which are not visible at 50 ms mixing time, for example for residues Q15 to F20. As these residues additionally do not show multiple peaks, this is the structurally most conserved part in the oligomer. Altogether, these characteristics made it possible to assign these resonances to the appropriate residue in the amino acid sequence.

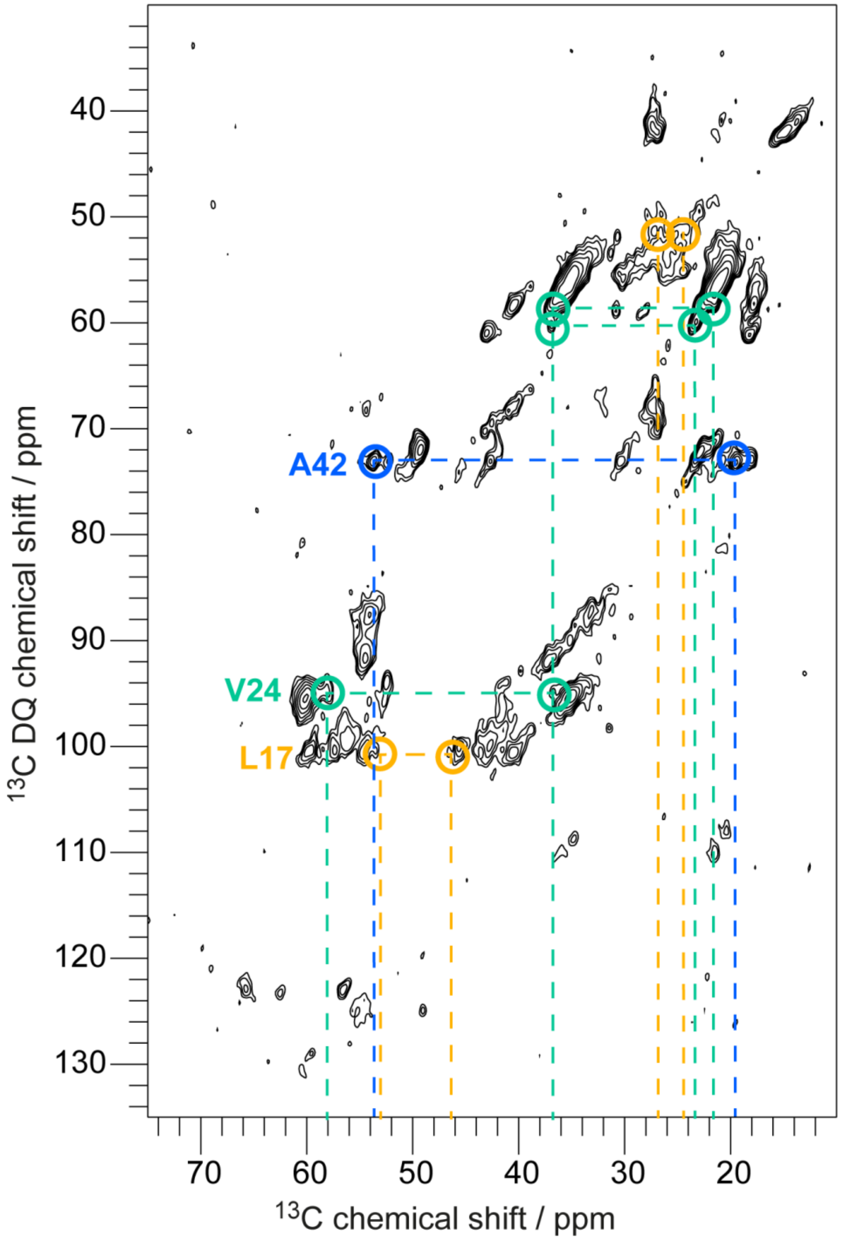

Figure S15. Double-quantum (DQ) correlation spectrum of huPrP(23-144)-Aβ* (* species is ^{13}C, ^{15}N uniformly labeled) with SPC5-recoupling, recorded at a temperature of ≈ 0 °C and a spinning frequency of 8 kHz. Three identified residues are shown exemplarily with colored circles and dashed lines: L17 (orange), V24 (green) and A42 (blue).

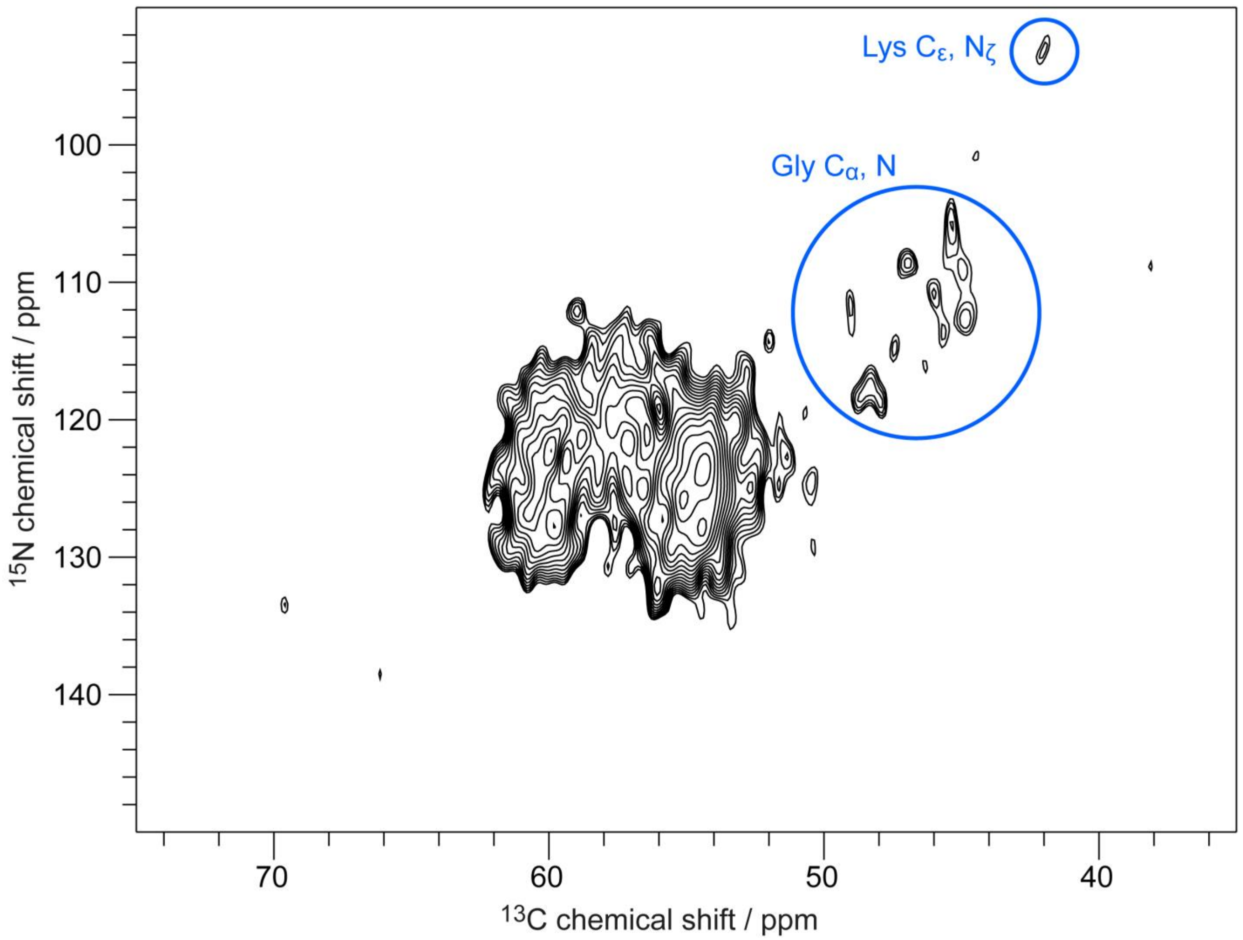

Figure S16. NCA spectrum of huPrP(23-144)-Aβ* (* species is ^{13}C, ^{15}N uniformly labeled), recorded at a temperature of ≈ 0 °C and a spinning frequency of 11 kHz. Blue circles indicate some identified amino acids.

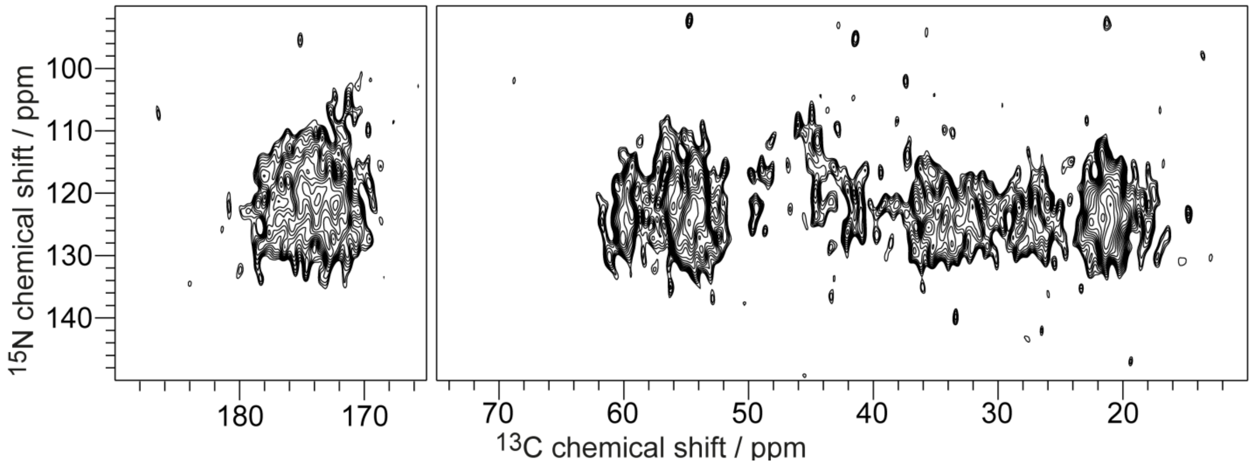

Figure S17. 2D NCACX spectrum of huPrP(23-144)-Aβ* (* species is ^{13}C, ^{15}N uniformly labeled), recorded at a temperature of ≈ 0 °C and a spinning frequency of 11 kHz.

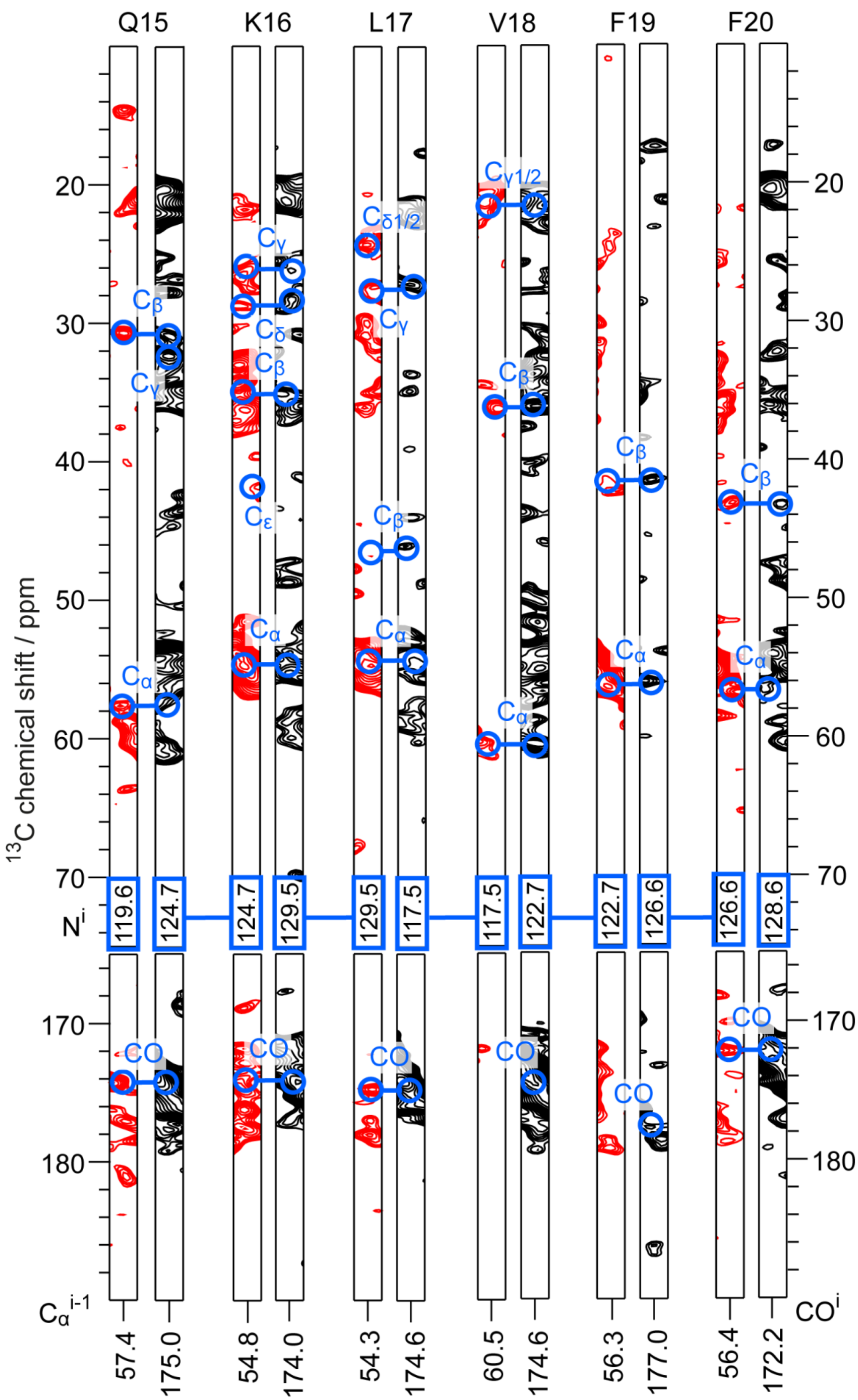
Q15
K16
L17
V18
F19
F20
13C chemical shift / ppm
Cβ
Cγ
Cα
CO
Cε
Cδ
Cδ1/2
Cγ1/2
Ni
119.6
124.7
124.7
129.5
129.5
117.5
117.5
122.7
122.7
126.6
126.6
128.6
Cαi-1
COi
57.4
175.0
54.8
174.0
54.3
174.6
60.5
174.6
56.3
177.0
56.4
172.2

Figure S18. Representative strip plot of huPrP(23-144)-Aβ* (* species is ^{13}C, ^{15}N uniformly labeled), 2D slices of 3D-NCACX (red) and 3D-NCOCX (black) spectra, recorded at a temperature of ≈ 0 °C, a spinning frequency of 11 kHz and subsequent DARR-mixing with 60 (NCACX) or 70 (NCOCX) ms mixing time. The sequential walk for residues Q15 to F20 is shown in blue.

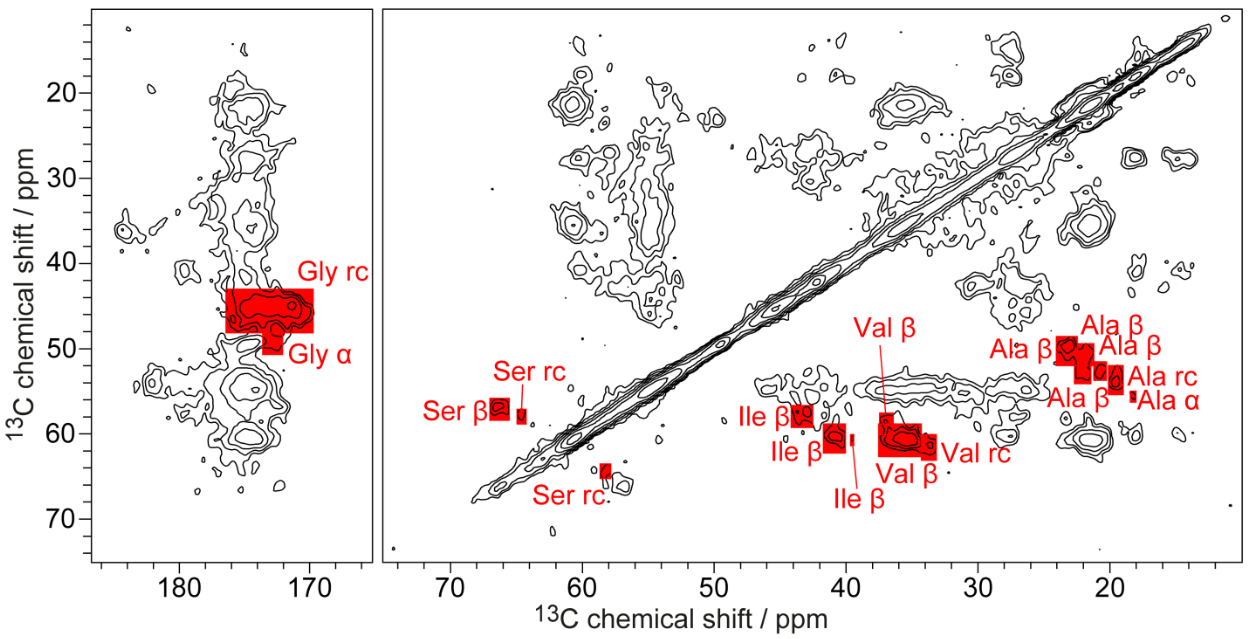

Figure S19. 2D PDSD spectrum of huPrP(23-144)-Aβ* (* species is ^{13}C, ^{15}N uniformly labeled), recorded at a temperature of ≈ 0 °C, a spinning frequency of 11 kHz and a mixing time of 50 ms. α-helical (labeled with 'α'), unstructured (labeled with 'rc', random coil) and β-strand like (labeled with 'β') conformations were integrated by the box sum method in Topspin (red boxes).

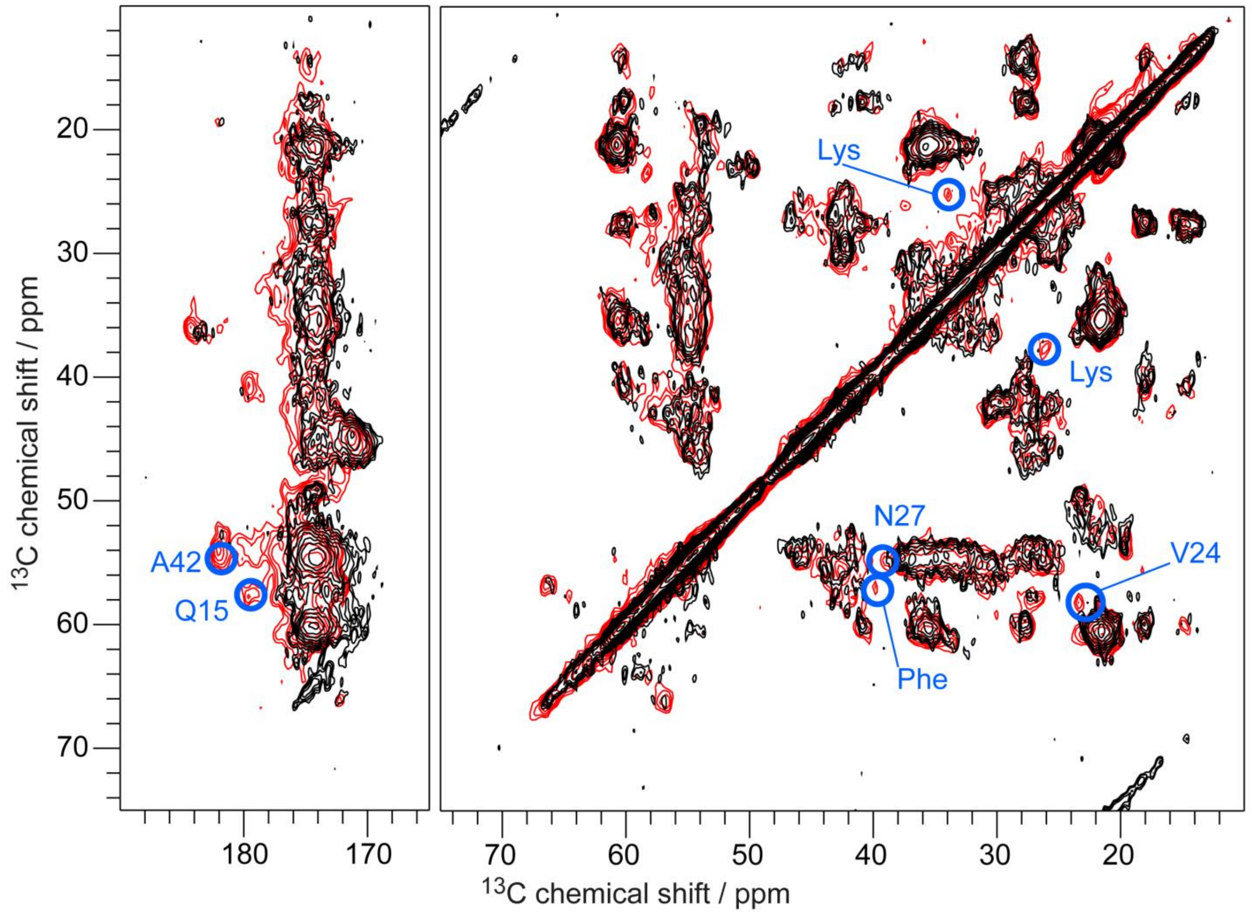

Figure S20. PDSD spectra of huPrP(23-144)-Aβ* (red) (* species is ^{13}C, ^{15}N uniformly labeled) and huPrP(23-144)$_{exc}$-Aβ* (black) ($_{exc}$ huPrP is in excess; * species is ^{13}C, ^{15}N uniformly labeled), recorded at a temperature of ≈ 0 °C, at a spinning frequency of 11 kHz and a mixing time of 50 ms at either a 600 MHz (huPrP(23-144)-Aβ*) or 800 MHz (huPrP(23-144)$_{exc}$-Aβ*) spectrometer. Blue circles highlight differences between the two samples.

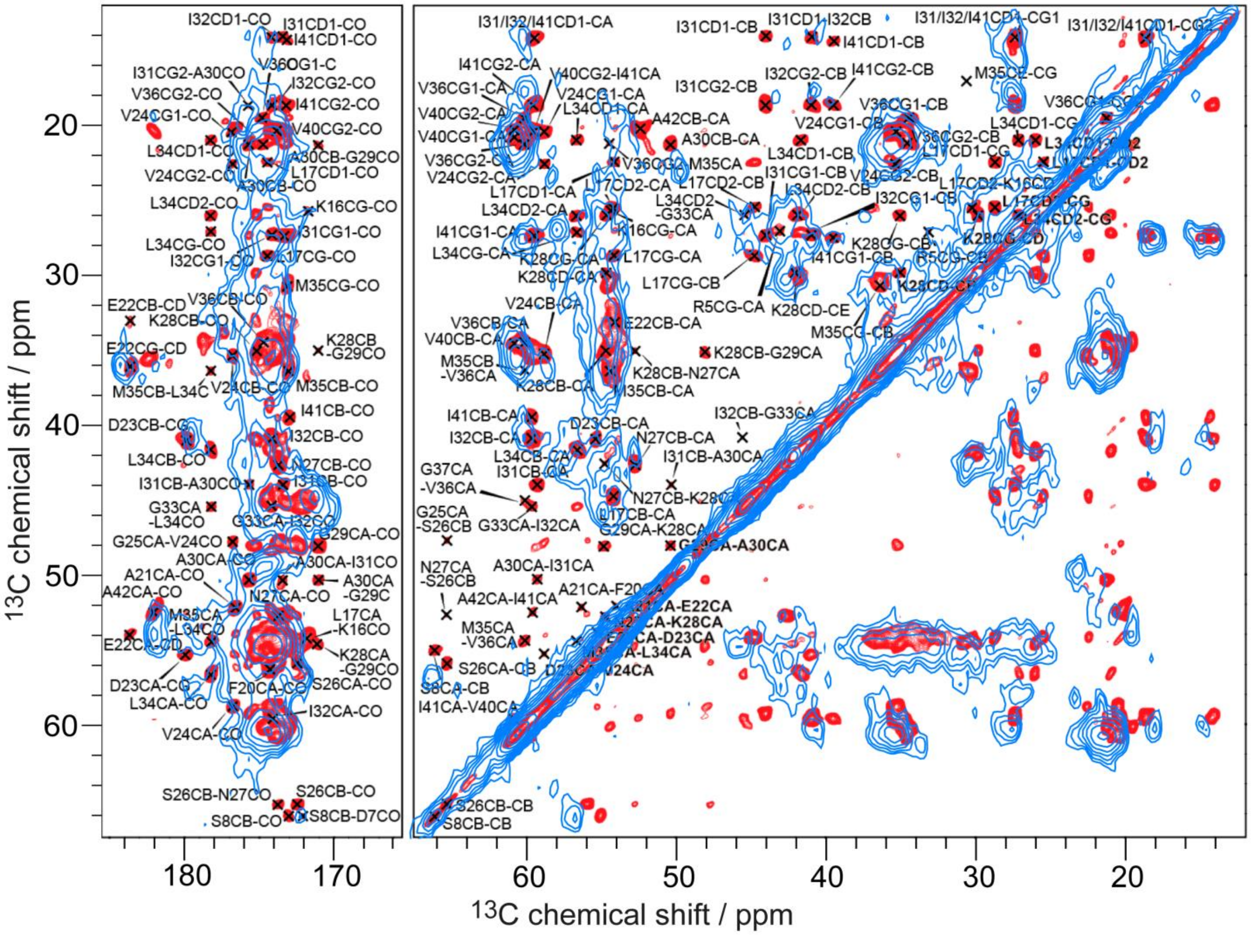

Figure S21. Overlay of a PDSD spectrum (blue) of huPrP(23-144)-Aβ* (* species is ^{13}C, ^{15}N uniformly labeled), recorded at a temperature of ≈ 0 °C, a spinning frequency of 11 kHz and a mixing time of 50 ms (same spectrum as in **Figure 5**), and a DARR spectrum (red) of the Aβ(1-42) fibril at pH 7.4 of Colvin et al. (98), recorded at a $\omega(^{1}H)/2\pi$ field strength of 800 MHz, a temperature of 277 K, a spinning frequency of 20 kHz and a mixing time of 80 ms. There is substantial signal overlap, but for the Aβ(1-42) fibril line widths are much smaller. Picture adapted with permission of ref. (98).

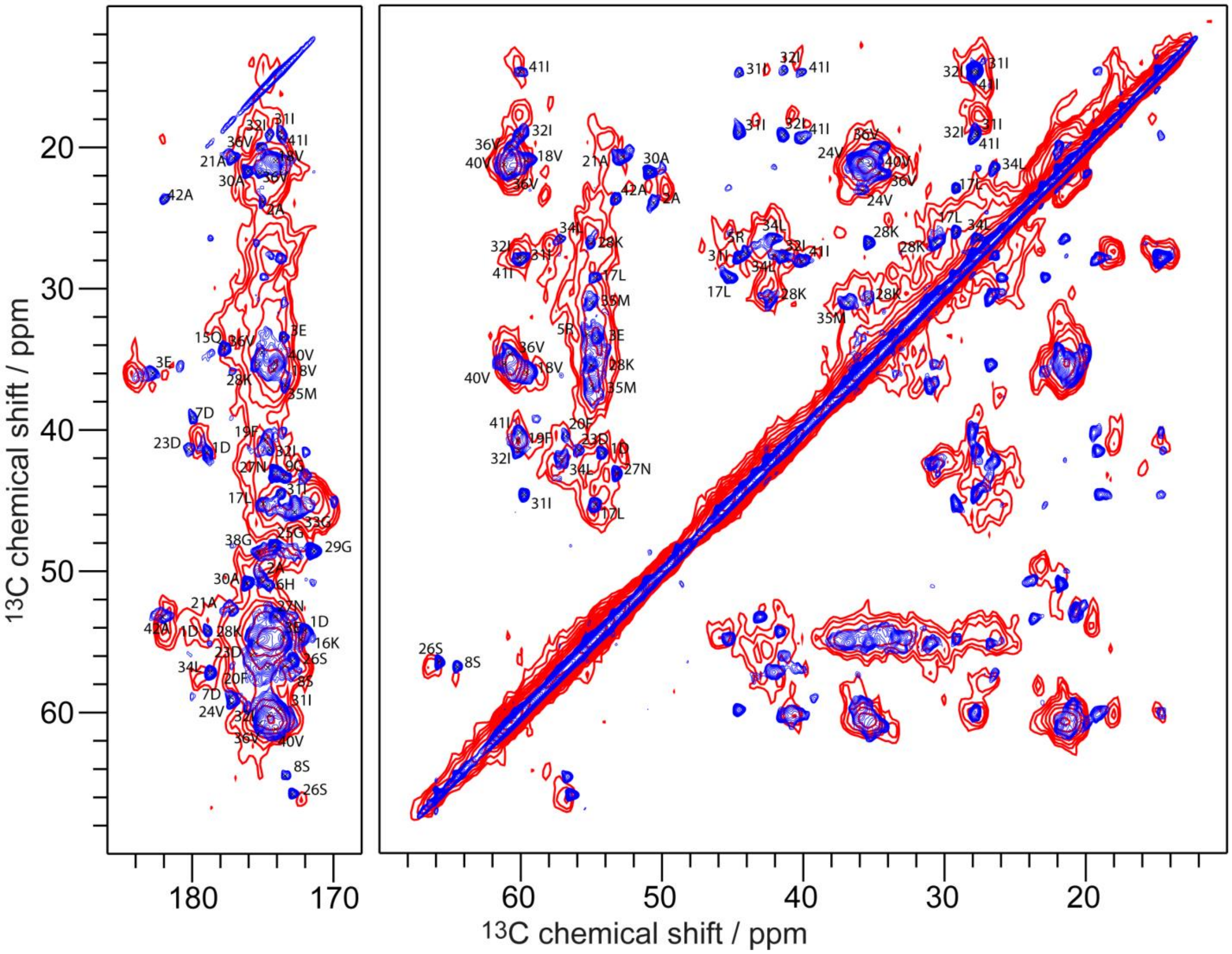

Figure S22. Overlay of a PDSD spectrum (red) of huPrP(23-144)-Aβ* (* species is ^{13}C, ^{15}N uniformly labeled), recorded at a temperature of ≈ 0 °C, a spinning frequency of 11 kHz and a mixing time of 50 ms (same spectrum as in **Figure 5**), and a DARR spectrum (blue) of the Aβ(1-42) fibril at pH 7.4 of Ravotti et al. (99), recorded at a magnetic field strength of 20.0 T, a spinning frequency of 17 kHz and a mixing time of 20 ms. There is substantial signal overlap, but for the Aβ(1-42) fibril line widths are much smaller. Picture adapted with permission of ref. (99).

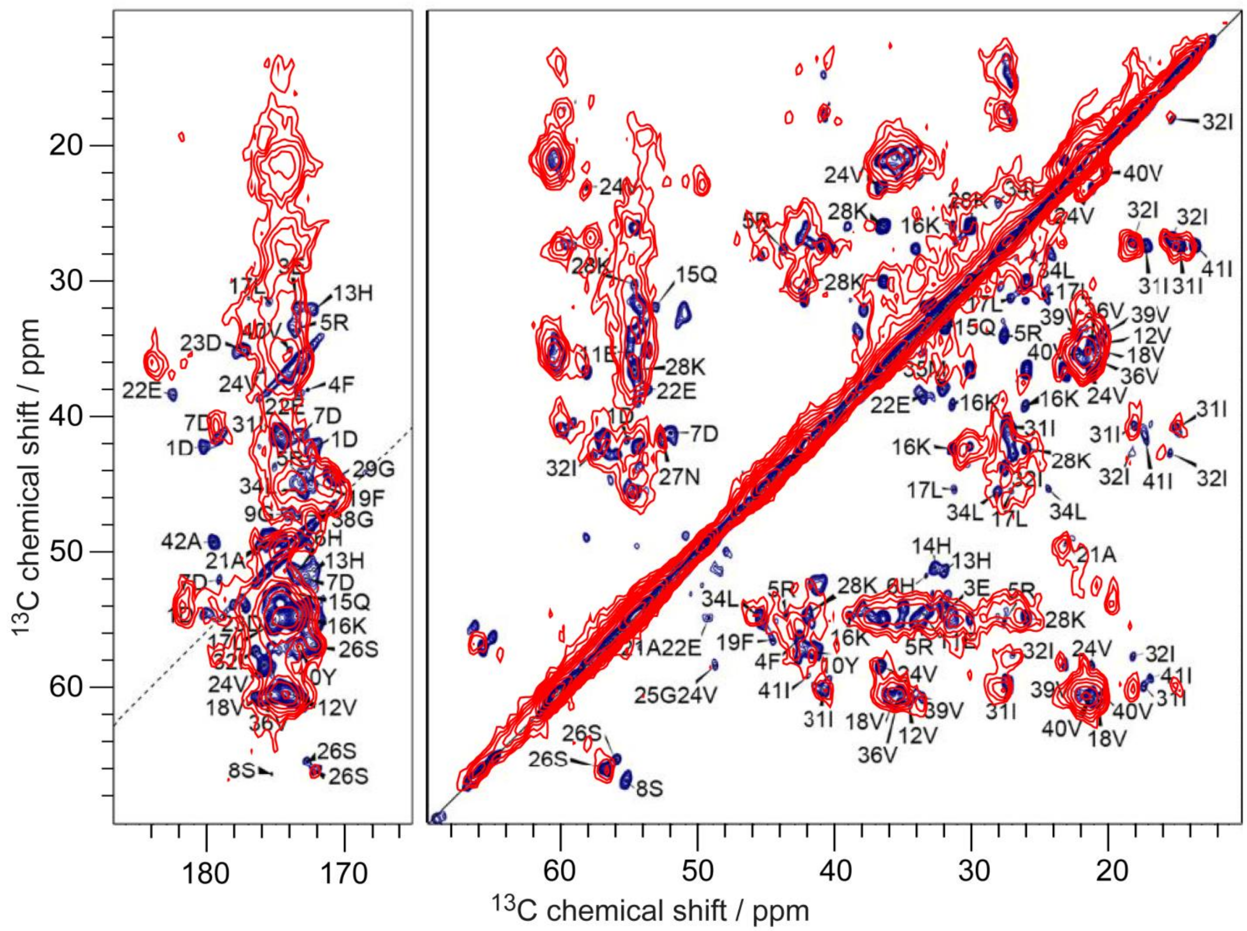

Figure S23. Overlay of two PDSD spectra of huPrP(23-144)-Aβ* (* species is ^{13}C, ^{15}N uniformly labeled), recorded at a temperature of ≈ 0 °C, a spinning frequency of 11 kHz and a mixing time of 50 ms (red, same spectrum as in **Figure 5**), and of the Aβ(1-42) fibril at pH 2 of Gremer et al. (blue) (47), recorded at a magnetic field strength of 18.8 T, a temperature of 0±5 °C, a spinning frequency of 12.5 kHz and a mixing time of 20 ms. There is substantial signal overlap, but for the Aβ(1-42) fibril line widths are much smaller. Picture adapted with permission of ref. (47).

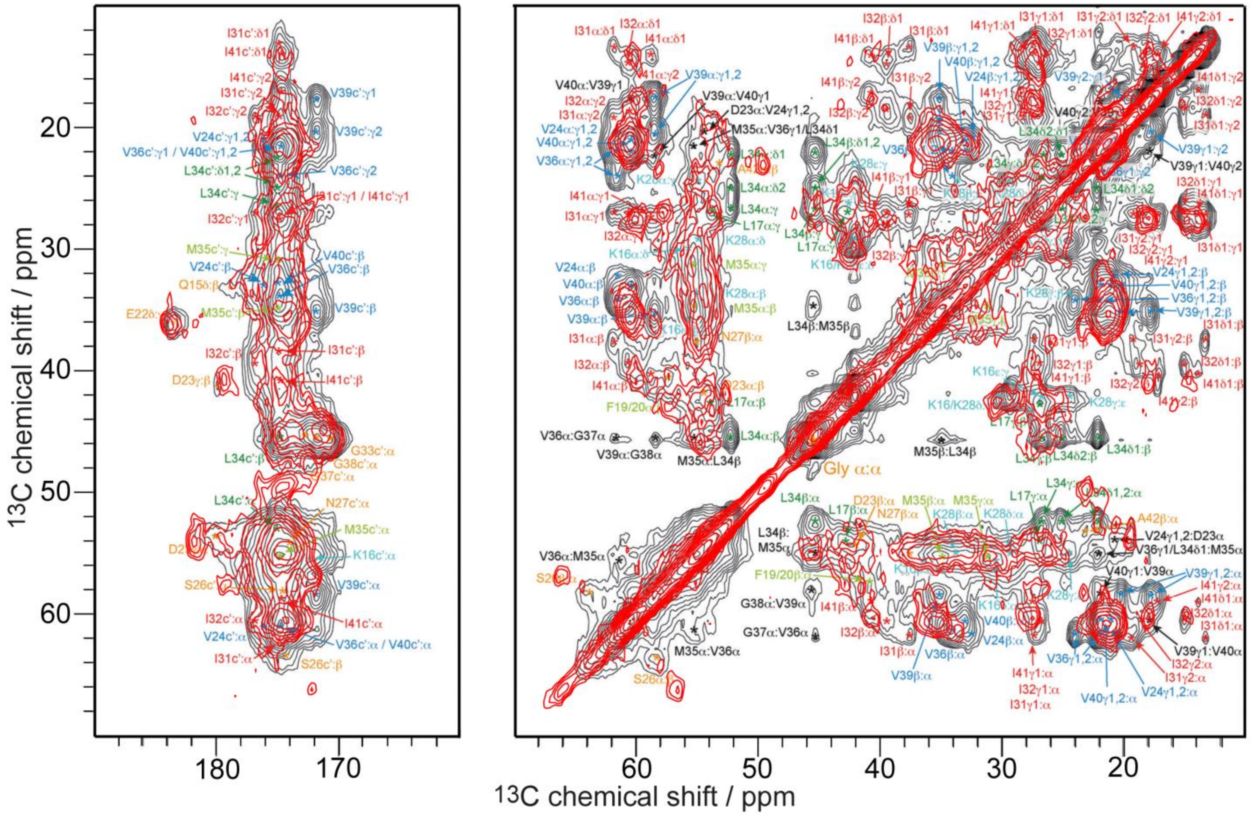

Figure S24. Overlay of a PDSD spectrum (red) of huPrP(23-144)-Aβ* (* species is ^{13}C, ^{15}N uniformly labeled), recorded at a temperature of ≈ 0 °C, a spinning frequency of 11 kHz and a mixing time of 50 ms (same spectrum as in **Figure 5**), and a DARR spectrum (black) of a hexameric Aβ42cc protofibril (mutated at A21C, A30C) of Lendel et al. (13), recorded at a $\omega(^{1}H)/2\pi$ field strength of 700 MHz, a temperature of ≈ 4 °C, a spinning frequency of 12 kHz and a mixing time of 50 ms. There is some signal overlap, although some peaks are not existing in the other Aβ species, but line widths are almost similar. Picture adapted with permission of ref. (13).

Table S1. Chemical shifts of unambiguously assigned resonances in huPrP(23-144)-Aβ* (* species is ^{13}C, ^{15}N uniformly labeled).

#	Amino acid	N (ppm)	CO (ppm)	CA (ppm)	CB (ppm)	CG1 (ppm)	CG2 (ppm)	CD1 (ppm)	CD2 (ppm)	CE1 (ppm)	CE2 (ppm)	CZ (ppm)	ND2 (ppm)	NE1 (ppm)	NE2 (ppm)	NZ (ppm)
1	D															
2	A															
3	E															
4	F															
5	R	122.4	174.4	54.5	30.3	28.1		42.2				-		-		
		126.7	-	53.3	30.9	25.0		43.7				165.0		67.7		
6	H	-	-	-	-	-			-	-					-	
7	D															
8	S															
9	G															
10	Y															
11	E															
12	V															
13	H	-	-	-	-	-			-	-					-	
14	H	-	-	-	-	-			-	-					-	
15	Q	119.6	175.0	57.4	30.5	32.2		179.9							124.3	
16	K	124.7	174.0	54.8	35.3	25.9		28.4		42.1						34.8
17	L	129.5	174.6	54.3	46.5	27.2		24.8	24.8							
18	V	117.5	174.6	60.5	35.5	21.2	21.2									
19	F	122.7	177.0	56.3	41.0	137.1		131.7	131.7	130.8	130.8	129.0				
20	F	126.6	172.2	56.4	43.5	137.3		131.8	131.8	129.9	129.9	127.5				
21	A															
22	E															
23	D	123.3	174.6	54.6	41.6	178.9										
24	V	122.1	176.6	58.2	36.8	23.2	21.6									
25	G	113.6	171.3	45.8												
26	S	119.5	172.2	56.6	66.2											
27	N	116.4	174.9	53.0	43.2	179.1							114.3			
		129.9	174.1	54.8	38.5	177.1							124.8			
28	K	119.1	176.8	54.4	33.7	25.1		28.8		42.2						32.9
29	G	116.7	172.6	49.2												
30	A	124.4	175.1	49.5	22.9											

31	I	124.1	173.9	57.7	43.0	27.0	18.1	15.4								
32	I	126.9	174.7	60.2	40.7	27.4	17.9	14.7								
33	G	119.1	173.8	48.6												
34	L															
35	M	-	-	-	-	-				-						
36	V															
37	G															
38	G															
39	V															
40	V	120.6	175.0	60.6	32.0	21.6	21.6									
41	I	122.3	175.3	60.2	39.4	27.5	17.9	13.9								
42	A	117.3	182.1	53.9	19.4											

Table S2. Overview of the performed solid-state NMR measurements.

experiment	description	dimen-sions	transfer	purpose	samples
INEPT (75)	insensitive nuclei enhanced by polarization transfer	1D	^{1}H to ^{13}C	to selectively excite mobile regions	huPrP(23-144)*-Aβ<br>huPrP(23-230)*-Aβ<br>huPrP(23-144)-Aβ*<br>huPrP(23-144)$_{exc}$-Aβ*
CP	cross polarization	1D	^{1}H to ^{13}C	to excite rigid parts	huPrP(23-144)*-Aβ<br>huPrP(23-230)*-Aβ<br>huPrP(23-144)-Aβ*<br>huPrP(23-144)$_{exc}$-Aβ*
			^{1}H to ^{15}N	to excite rigid parts	huPrP(23-144)*-Aβ<br>huPrP(23-144)-Aβ*<br>huPrP(23-144)$_{exc}$-Aβ*
PDSD (76)	proton driven spin diffusion	2D	^{13}C to ^{13}C	to excite all correlations	huPrP(23-144)*-Aβ<br>huPrP(23-230)*-Aβ<br>huPrP(23-144)-Aβ*<br>huPrP(23-144)$_{exc}$-Aβ*
DARR	dipolar assisted rotational resonance	2D	^{13}C to ^{13}C	to excite all correlations	huPrP(23-144)-Aβ*
DQ-SPC5 (77)	double quantum excitation with SPC5-recoupling	2D	^{13}C to ^{13}C	to excite only one-bond correlations	huPrP(23-144)*-Aβ<br>huPrP(23-144)-Aβ*
NCA (78)	with SPECIFIC-CP	2D	^{15}N to ^{13}Cα	frequency selective polarization transfers from ^{15}N to ^{13}Cα	huPrP(23-144)*-Aβ<br>huPrP(23-144)-Aβ*
NCACX (78)	with SPECIFIC-CP	2D and 3D	^{15}N to ^{13}Cα to ^{13}C$_{sidechain}$	frequency selective polarization transfers from ^{15}N to ^{13}Cα, subsequent DARR mixing to distribute magnetization to side chains	huPrP(23-144)-Aβ*
NCOCX (78)	with SPECIFIC-CP	3D	^{15}N to ^{13}CO to ^{13}C$_{sidechain}$	frequency selective polarization transfers from ^{15}N to ^{13}CO, subsequent DARR mixing to distribute magnetization to side chains	huPrP(23-144)-Aβ*

Table S3. Experimental parameters for solid-state NMR measurements of sample huPrP(23-144)*-Aβ (* species is ^{13}C, ^{15}N uniformly labeled).

**Sample huPrP(23-144)*-Aβ**						
	HC CP	HN CP	INEPT	PDSD	SPC5_2	NCA
	1D	1D	1D	2D	2D	2D
Mixing time (ms)	-	-	-	1) 20<br>2) 30<br>3) 50<br>4) 100<br>5) 200<br>6) 200 R^2	-	-
^1H frequency (MHz)	600	600	600	600	600	600
MAS (kHz)	11	11	4	1) & 2) & 3) & 4) & 5) 11<br>6) 9.375	8	11
VT gas temperature (°C)	-10	-16	RT	1) & 3) & 4) -10<br>2) & 5) & 6) -16	-16	-16
Transfer 1	HC CP	HN CP	INEPT	HC CP	HC CP	HN CP
Carrier (ppm)	70.525	119.979	70.525	1) & 3) & 4) 70.525<br>2) & 5) & 6) 90.046	70.525	119.973
Duration of 1st transfer (μs)	200	600	-	1) & 3) & 4) 200<br>2) & 5) & 6) 350	300	600
Transfer 2	-	-	-	-	SPC5	N-CA CP
Carrier (ppm)	-	-	-	-	60.579	57.058
Duration of 2nd transfer (μs)	-	-	-	-	-	1800
^{13}C rf field (kHz)	-	-	-	-	-	8.205
^{15}N rf field (kHz)	-	-	-	-	-	18.803
t_1 increments	454	136	454	1) & 3) & 4) 454<br>2) & 5) & 6) 379	454	366
t_1 spectral width (kHz)	37.879	13.587	37.879	37.879	37.879	30.488
t_2 increments	-	-	-	1) & 5) 180<br>2) 260<br>3) & 4) 128<br>6) 190	240	22
t_2 spectral width (kHz)	-	-	-	1) & 3) & 4) 29.996<br>2) & 5) 33.003<br>6) 37.700	40.000	3.600
Number of scans	256	2000	256	1) 128<br>2) & 3) & 4) 320	592	1600

				5) 216<br>6) 568		
Duration (h)	0.15	1.7	0.15	1) 26.25<br>2) 95<br>3) 47.25<br>4) 48.5<br>5) 43.75<br>6) 133.5	160.5	40

Table S4. Experimental parameters for solid-state NMR measurements of sample huPrP(23-230)*-Aβ (* species is ^{13}C, ^{15}N uniformly labeled).

**Sample huPrP(23-230)*-Aβ**								
	^{13}C DE	^{13}C DE	^{13}C DE	HC CP	HC CP	HC CP	INEPT	PDSD
	1D	1D	1D	1D	1D	1D	1D	2D
Mixing time (ms)	-	-	-	-	-	-	-	1) 30<br>2) 50
^{1}H frequency (MHz)	600	600	600	600	600	600	600	600
MAS (kHz)	11	11	11	11	11	11	11	11
VT gas temperature (°C)	20	0	-20	20	0	-20	20	1) -10<br>2) 0
Transfer 1	-	-	-	HC CP	HC CP	HC CP	INEPT	HC CP
Carrier (ppm)	79.967	79.967	79.967	79.967	79.967	79.967	79.967	79.967
Duration of 1st transfer (μs)	-	-	-	200	200	200	-	200
t_1 increments	750	750	750	750	750	750	750	750
t_1 spectral width (kHz)	62.500	62.500	62.500	62.500	62.500	62.500	62.500	62.500
t_2 increments	-	-	-	-	-	-	-	1) 140<br>2) 134
t_2 spectral width (kHz)	-	-	-	-	-	-	-	36.199
Number of scans	2304	1021	2400	512	512	512	5659	1) 1267<br>2) 272
Duration (h)	13	5.75	13.5	0.29	0.29	0.29	3.25	1) 201.75<br>2) 42

Table S5. Experimental parameters for solid-state NMR measurements of sample huPrP(23-144)-Aβ* (* species is ^{13}C, ^{15}N uniformly labeled).

**Sample huPrP(23-144)-Aβ***										
	HC CP	HN CP	INEPT	PDSD	DARR	SPC5_2	NCA	NCACX DARR	NCACX DARR	NCOCX DARR
	1D	1D	1D	2D	2D	2D	2D	2D	3D	3D
Mixing time (ms)	-	-	-	1) 10<br>2) 30<br>3) 50<br>4) 50<br>5) 50 R^2<br>6) 100<br>7) 200<br>8) 200 R^2<br>9) 300	30	-	-	60	60	70
^{1}H frequency (MHz)	600	600	600	1) & 2) & 3) & 6) & 7) & 8) & 9) 600<br>4) & 5) 800	600	600	600	600	600	600
MAS (kHz)	11	11	8	1) & 2) & 3) & 4) & 6) & 7) & 9) 11<br>5) 12.5<br>8) 9.375	11	8	11	11	11	11
VT gas temperature (°C)	-10	-10	10	-10	-10	-10	-10	-10	-10	-10
Transfer 1	HC CP	HN CP	INEPT	HC CP	HC CP	HC CP	HN CP	HN CP	HN CP	HN CP
Carrier (ppm)	70.978	120.159	70.978	1) & 2) & 3) & 6) & 7) & 8) & 9) 70.978<br>4) & 5) 70.307	70.978	1) 70.525<br>2) 70.153	119.979	119.979	119.979	119.979
Duration of 1st transfer (μs)	400	500	-	400	400	1) 400<br>2) 500	900	900	900	900
Transfer 2	-	-	-	-	-	SPC5	N-CA CP	N-CA CP	N-CA CP	N-CO CP
Carrier (ppm)	-	-	-	-	-	1) 67.210<br>2) 23.735	57.057	57.057	57.057	174.950

Duration of 2nd transfer (μs)	-	-	-	-	-	-	1600	1600	1600	1800
^{13}C rf field (kHz)	-	-	-	-	-	-	17.806	17.806-18.824	18.824-19.078	38.156
^{15}N rf field (kHz)	-	-	-	-	-	-	28.490	28.490-29.304	28.490-29.304	27.676
t_1 increments	454	181	454	1) & 2) & 3) & 6) & 7) & 8) & 9) 454<br>4) & 5) 1250	454	1) 454<br>2) 536	366	536	402	536
t_1 spectral width (kHz)	37.879	18.116	37.879	1) & 2) & 3) & 6) & 7) & 8) & 9) 37.879<br>4) & 5) 104.167	37.879	1) 37.879<br>2) 44.643	30.488	44.643	44.643	59.524
t_2 increments	-	-	-	1) & 2) & 3) & 6) & 7) & 9) 200<br>4) & 5) 264<br>8) 225	200	1) & 2) 240	22	22	18	12
t_2 spectral width (kHz)	-	-	-	1) & 2) & 3) & 6) & 7) & 9) 33.003<br>4) & 5) 44.000<br>8) 37.700	33.003	1) & 2) 40.000	3.600	3.600	3.600	2.400
t_3 increments	-	-	-	-	-	-	-	-	20	13
t_3 spectral width (kHz)	-	-	-	-	-	-	-	-	5.100	3.300
Number of scans	128	2000	256	1) 304<br>2) & 3) & 5) & 7) 288<br>4) 224<br>6) 368<br>8) 320<br>9) 352	288	1) 320<br>2) 496	864	1696	736	1072
Duration (h)	0.08	1.75	0.15	1) 69<br>2) 66<br>3) & 4) 66.5	66	1) 86.75<br>2) 133.25	21.25	159.25	612	387.75

	5) & 6) 87<br>7) 71.25<br>8) 89<br>9) 91

Table S6. Experimental parameters for solid-state NMR measurements of sample huPrP(23-144)$_{exc}$-Aβ* (* species is ^{13}C, ^{15}N uniformly labeled).

**Sample huPrP(23-144)$_{exc}$-Aβ***				
	HC CP	HN CP	INEPT	PDSD
	1D	1D	1D	2D
Mixing time (ms)	-	-	-	1) 50<br>2) 50 (after 4 months)
^{1}H frequency (MHz)	800	800	800	800
MAS (kHz)	5	11	8	11
VT gas temperature (°C)	-3	-10	10	-10
Transfer 1	HC CP	HN CP	INEPT	HC CP
Carrier (ppm)	70.183	124.191	70.000	70.183
Duration of 1sttransfer (μs)	600	600	-	600
t_1 increments (half of STATES-TPPI)	778	949	778	778
t_1 spectral width (kHz)	52.083	59.524	52.083	52.083
t_2 increments (half of STATES-TPPI)	-	-	-	256
t_2 spectral width (kHz)	-	-	-	50.505
Number of scans	256	2048	1024	432
Duration (h)	0.15	3	0.5	127.75

Table S7. Overview of the performed solution NMR measurements.

experiment	description	dimensions	transfer	purpose	Samples
HSQC	heteronuclear single-quantum coherence	2D	^{1}H to ^{15}N and back	amide groups	huPrP(23-144) monomers
HNCO	triple resonance	3D	^{1}H to ^{15}N to ^{13}CO and back	resonance assignment	huPrP(23-144) monomers
HNCACB	triple resonance	3D	^{1}H to ^{15}N to ^{13}Cα to ^{13}Cβ and back	resonance assignment	huPrP(23-144) monomers
BEST-TROSY-(H)N(COCA)NH	triple resonance	3D	^{1}H to ^{15}N to ^{13}CO to ^{13}Cα to ^{15}N to ^{1}H	resonance assignment	huPrP(23-144) monomers
TOCSY	total correlated spectroscopy	2D	^{13}C to ^{13}C	to excite all correlations	huPrP(23-144) monomers Aβ monomers
